# A supramolecular complex of palmitoylated Orai C-terminal peptide and cyclodextrin targets the plasma membrane to reduce store-operated calcium channel activity

**DOI:** 10.1039/d6ma00756b

**Published:** 2026-08-21

**Authors:** Juan Toledo-Marcos, Bruno Di Geronimo, Sanja Curcic, Viktor Farkas, Pedro A Sánchez-Murcia, Rainer Schindl, Bernadett Bacsa, Kata Horváti

**Affiliations:** a Division of Medical Physics and Biophysics, Gottfried Schatz Research Center, Medical University of Graz Neue Stiftingtalstrasse 6 8010 Graz Austria bernadett.bacsa@medunigraz.at; b School of Chemistry and Biochemistry, Georgia Institute of Technology 901 Atlantic Drive NW Atlanta 30332-0400 Georgia; c HUN-REN–ELTE Protein Modeling Research Group, Eötvös Lorand University Pázmány Péter stny. 1/a, H-1117, Budapest Hungary; d Laboratory of Computer-Aided Molecular Design, Division of Medicinal Chemistry, Otto-Loewi Research Center, Medical University of Graz Neue Stiftingtalstrasse 6, 8010 Graz Austria; e MTA–HUN-REN TTK Lendület “Momentum” Peptide-Based Vaccines Research Group, Institute of Materials and Environmental Chemistry, HUN-REN Research Centre for Natural Sciences Magyar Tudósok Krt. 2 H-1117 Budapest Hungary horvati.kata@ttk.hu; f BioTechMed-Graz, Austria Auenbruggerplatz 30 8036 Graz Austria

## Abstract

Palmitoylation is a widely employed strategy for anchoring peptides to lipid membranes, but it can also lead to uncontrolled self-assembly. The lipid moieties can promote clustering into heterodispersed aggregates that distribute unevenly, thereby reducing accessibility and inducing undesired uptake mechanisms that limit the functional performance of peptides. Controlling this lipid-driven aggregation remains a key challenge for membrane-active peptide systems. Here, we introduce a supramolecular entrapment strategy for controlling the assembly and membrane insertion of bioactive lipopeptides. We synthesize palmitoylated Orai3-derived peptides and use randomly methylated α-cyclodextrin (RAMEA) to complex the fatty acid chain through a host–guest inclusion mechanism, thereby effectively shielding its intermolecular hydrophobic interactions. In addition, we incorporate an octaethylene glycol (PEG8) spacer to improve peptide flexibility and access to its molecular target at the membrane interface. Biophysical characterization and molecular dynamics simulations show that the complex formation between the cyclodextrin and the lipopeptide yields an ultralow-sized molecular dispersion system with reduced hydrophobic exposure and aggregation propensity. In cell-based systems, this supramolecular organization enhances membrane localization and reduces endosomal internalization, while the PEG8 spacer further facilitates intracellular target engagement. This strategy was evaluated in the context of store-operated calcium (SOC) channel regulation, a well-characterized membrane-associated system that critically depends on precise spatial organization of interacting components, namely STIM1–Orai1 at the junctions between plasma and endoplasmic reticulum membranes. In this context, we show that this lipopeptide–cyclodextrin multiplex enables inhibition of SOC channel activity. Overall, this work demonstrates that our lipopeptide–RAMEA inclusion complexation strategy provides a general approach to control palmitoylation-driven lipopeptide aggregation and establishes a broadly applicable design principle for tuning the performance of membrane-associated peptide systems.

## Abstract 2

A lipopeptide–cyclodextrin inclusion strategy that controls palmitoylation-driven aggregation, enhances membrane localization and target engagement, providing a general design principle for the optimization of bioactive peptides.

## Graphical abstract

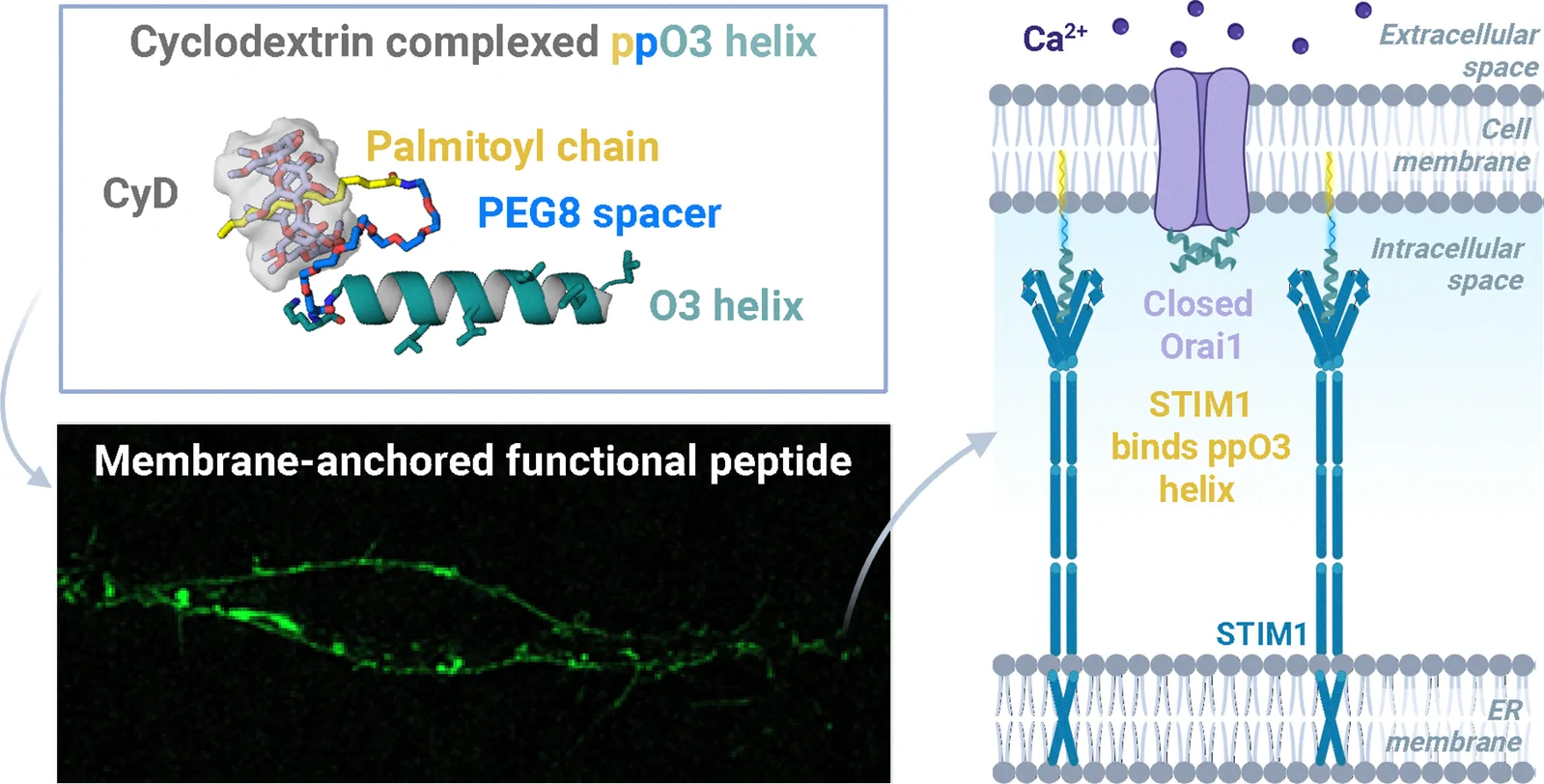

## 1. Introduction

Calcium ions (Ca^2+^) are key intracellular messengers regulating numerous physiological processes.^1,2^ One major Ca^2+^ entry pathway, activated by the depletion of intracellular Ca^2+^ stores, is known as the store-operated Ca^2+^ entry (SOCE) route.^3–6^ The key components of SOCE, which assemble at the endoplasmic reticulum–plasma membrane (ER–PM) junction, are two proteins: the ER-resident Ca^2+^ sensor stromal interaction molecule (STIM) and the plasma membrane Orai Ca^2+^ channel. Upon ER Ca^2+^ depletion, STIM1 senses the decrease in ER Ca^2+^ concentration, binds to Orai1, and opens this highly Ca^2+^-selective channel.^7^ Direct interaction of STIM1 with the cytosolic C-terminus of Orai1 is required for channel gating and Ca^2+^ influx.^3,7^

SOC channels are implicated in the pathophysiology of pulmonary^8–10^ and cardiovascular diseases,^11–13^ hypocalcemia,^14^ neurological disorders,^15,16^ and cancers.^17,18^ In the immune system, specifically STIM1/Orai1 channels are essential for activation of T lymphocytes and mast cells^19^ and represent potential therapeutic targets for inflammatory and autoimmune diseases.^20,21^ Despite their clinical relevance, only a few Orai channel inhibitors have advanced to phase II clinical trials,^22^ indicating the need for alternative strategies. In addition to the physiologically and functionally best characterized STIM1 and Orai1, the Orai channel family consists of two additional isoforms, Orai2 and Orai3. Among the three isoforms, Orai3 contains the highest affinity for STIM1 interaction.^23^ Peptides derived from the Orai3 C-terminus, acting as Orai dominant-negative peptides, have been shown to interfere with STIM1-Orai coupling, thereby reducing Ca^2+^ influx and tumour cell proliferation.^24^ In a related approach, Orai3 C-terminal fragments were genetically expressed and tethered to the inner leaflet of the plasma membrane, where they interacted with the STIM1 Orai-activating region (SOAR). Membrane anchoring of these intracellularly expressed peptides competitively disrupted STIM1-Orai coupling and reduced endogenous Ca^2+^ entry, demonstrating that membrane targeting of the Orai3 C-terminus is sufficient to modulate SOC channel activity.^25,26^

However, strategies based on intracellular expression are not suitable for therapeutic application, and the use of synthetic peptides is limited by low bioavailability and targeting capacity.^27,28^ Due to their size, charge, and tendency to oligomerize, peptides are predominantly internalized *via* endocytic pathways.^29^ Failure to escape from endosomes leads to degradation following endosome–lysosome fusion, preventing efficient access to intracellular targets. To address these limitations, alternative delivery strategies are required to enhance cytoplasmic access.

The clinical utility of palmitoylated peptides spans cancer therapy, cardiovascular and metabolic diseases, neurodegenerative disorders, and immune modulation, with growing evidence supporting their role as vaccine adjuvants and immune checkpoint regulators.^30–33^ However, the self-assembly of lipopeptides—which can result in the formation of aggregates or highly structured fibrillar forms—limits their application, because the stability of the solutions is often insufficient, and the large-sized associates preclude the option of sterile filtration.^34^ Therefore, spacers and various excipients are used by the pharmaceutical industry, among which cyclodextrins (CyDs) have proven to be successful, as they can produce filterable, monodisperse systems from heterogeneous suspensions containing apolar or amphiphilic active ingredients.^35,36^ CyDs are cyclic oligosaccharides renowned for their ability to form host–guest inclusion complexes with a wide range of molecules, including peptides and lipopeptides. Their unique toroidal structure, featuring a hydrophobic cavity and hydrophilic exterior, underpins their supramolecular chemistry and broad utility in drug delivery, nanomedicine, and materials science. Randomly methylated α-cyclodextrin (RAMEA) is a chemically modified derivative of α-cyclodextrin in which methyl groups are randomly substituted on the glucose units, capable of forming supramolecular complexes with both saturated and unsaturated fatty acids.^37^

To extend this strategy to SOC channels, a peptide derived from the Orai3 C-terminus was synthesized and modified with palmitic acid either directly or *via* an octaethylene glycol (PEG8) linker. The lipid moiety promotes membrane association, whereas the PEG8 linker provides conformational flexibility and may facilitate target engagement. To minimize aggregation and enable controlled membrane delivery, the palmitoylated Orai3 peptides were complexed with CyD. This design is expected to position the peptide in close proximity to STIM1 at ER–PM junctions, enabling competition with the Orai1 C-terminus and thereby interfering with STIM1-Orai coupling and Ca^2+^ influx through Orai1 channels.

## 2. Results and discussion

### 2.1. Synthesis and characterisation of Orai3 peptides and lipopeptides

**Scheme 1:**
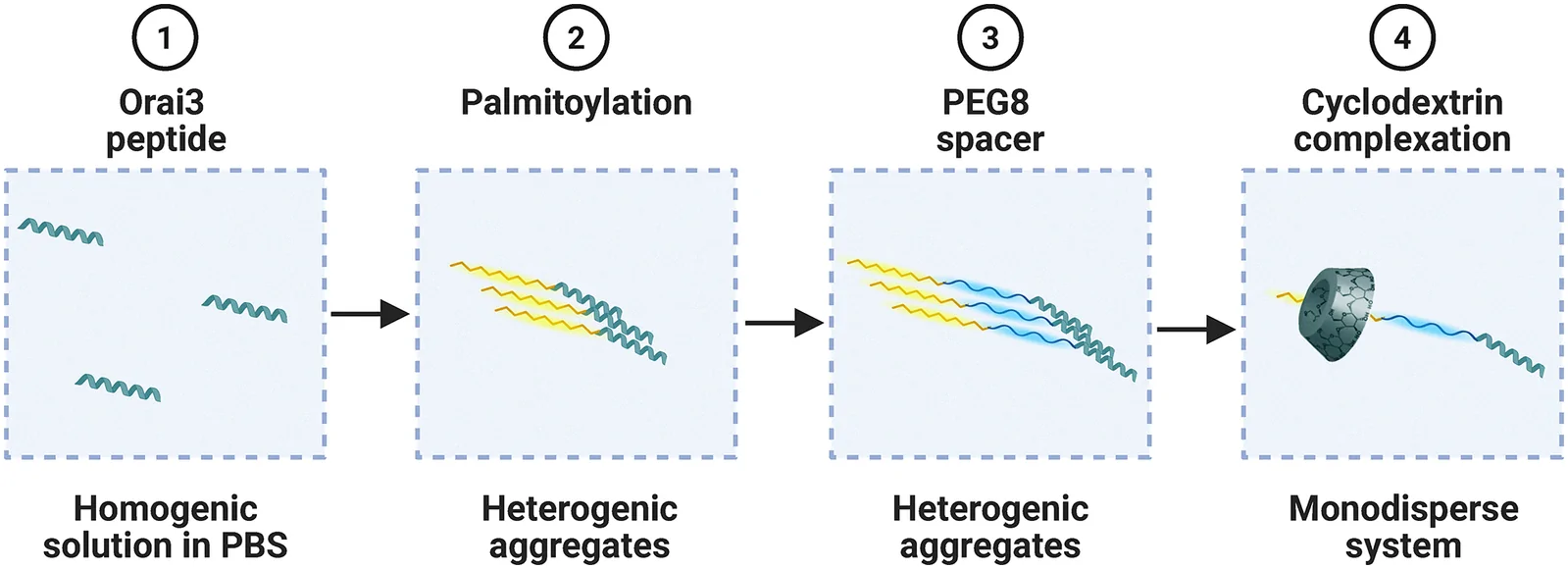
Flowchart of the peptide modification process and the behaviour in PBS (pH = 7.4).

Derivatives of the Orai3 C-terminal peptide (O3), shown in Table 1, were prepared using our further developed flow peptide synthesis method, which employs standard HPLC modules.^38–40^ Palmitic acid was attached to the N-terminus of the O3 peptide through an additional Lys residue (pO3). As a spacer, an octaethylene glycol (PEG8) molecule was inserted between the fatty acid and the peptide chain (ppO3, Scheme 1).

**Table 1:** Analytical characteristics of the peptides

Name	Sequence	*R*_t_ a (min)	*M* (mo) meas.^c^	*M* (mo) calc.^d^	*M* (av) calc.^d^	Mass error^e^ (ppm)
O3	H-**DRYKQELEELNRLQGELQAV**-NH_2_	12.4	2429.2719	2429.2663	2430.7031	2.31
Cf-O3	Cf-**DRYKQELEELNRLQGELQAV**-NH_2_	13.5/13.7^b^	2787.3205	2787.3140	2789.0099	2.33
pO3	CH_3_-(CH_2_)_14_-C(O)-**KDRYKQELEELNRLQGELQAV**-NH_2_	16.5	2795.5882	2795.5909	2797.2908	0.97
p(Cf)O3	CH_3_-(CH_2_)_14_-C(O)-**K**(Cf)**DRYKQELEELNRLQGELQAV**-NH_2_	17.8	3153.6346	3153.6386	3155.5976	1.27
ppO3	CH_3_-(CH_2_)_14_-C(O)-NH-CH_2_-CH_2_-(O-CH_2_-CH_2_)_7_-O-CH_2_-CH_2_-C(O)-**KDRYKQELEELNRLQGELQAV**-NH_2_	17.9	3218.8246	3218.8377	3220.7948	4.07
pp(Cf)O3	CH_3_-(CH_2_)_14_-C(O)-NH-CH_2_-CH_2_-(O-CH_2_-CH_2_)_7_-O-CH_2_-CH_2_-C(O)-**K**(Cf)**DRYKQELEELNRLQGELQAV**-NH_2_	18.8	3576.8818	3576.8855	3579.1016	1.03

a Retention time on a Phenomenex Jupiter C12 column, gradient: 5–100% B, 20 min. According to the RP-HPLC analysis, the purity of the peptides was always above 95%.

b In the case of the Cf-O3 peptide, the two retention time values correspond to the 5(6) isomers of carboxyfluorescein, eluted as two peaks on the HPLC chromatogram.

c Monoisotopic molecular mass measured on a Thermo Scientific Q Exactive Focus Hybrid Quadrupole-Orbitrap Mass Spectrometer.

d Monoisotopic and average masses, calculated using the Chemicalize program (https://chemicalize.com).

e Mass error was calculated as |(*M*(mo)measured − *M*(mo)calculated)/*M*(mo)calculated|.

Since fluorescently labelled derivatives were also required for cellular imaging, we prepared analogues in which 5(6)-carboxyfluorescein (Cf) was coupled to a lysine side chain. All peptides were produced with an RP-HPLC purity of at least 95%. Table 1 summarizes the analytical characteristics of the compounds, while the high-resolution mass spectra and chromatograms are shown in the SI (Fig. S1–S7). Since potential hemolytic activity and cytotoxicity are common concerns with lipopeptides, we used standard viability assays to determine the concentration range within which the peptides can be safely tested *in vitro* (Fig. S9). Based on the results obtained, all peptides can be used at concentrations up to 100 µM, with the exception of pO3, for which the viability of treated HEK293 cells was 65% at 100 µM but 100% at 50 µM.

### 2.2. Cyclodextrin aids solubilization of Orai3-derived lipopeptides

**Fig. 1:**
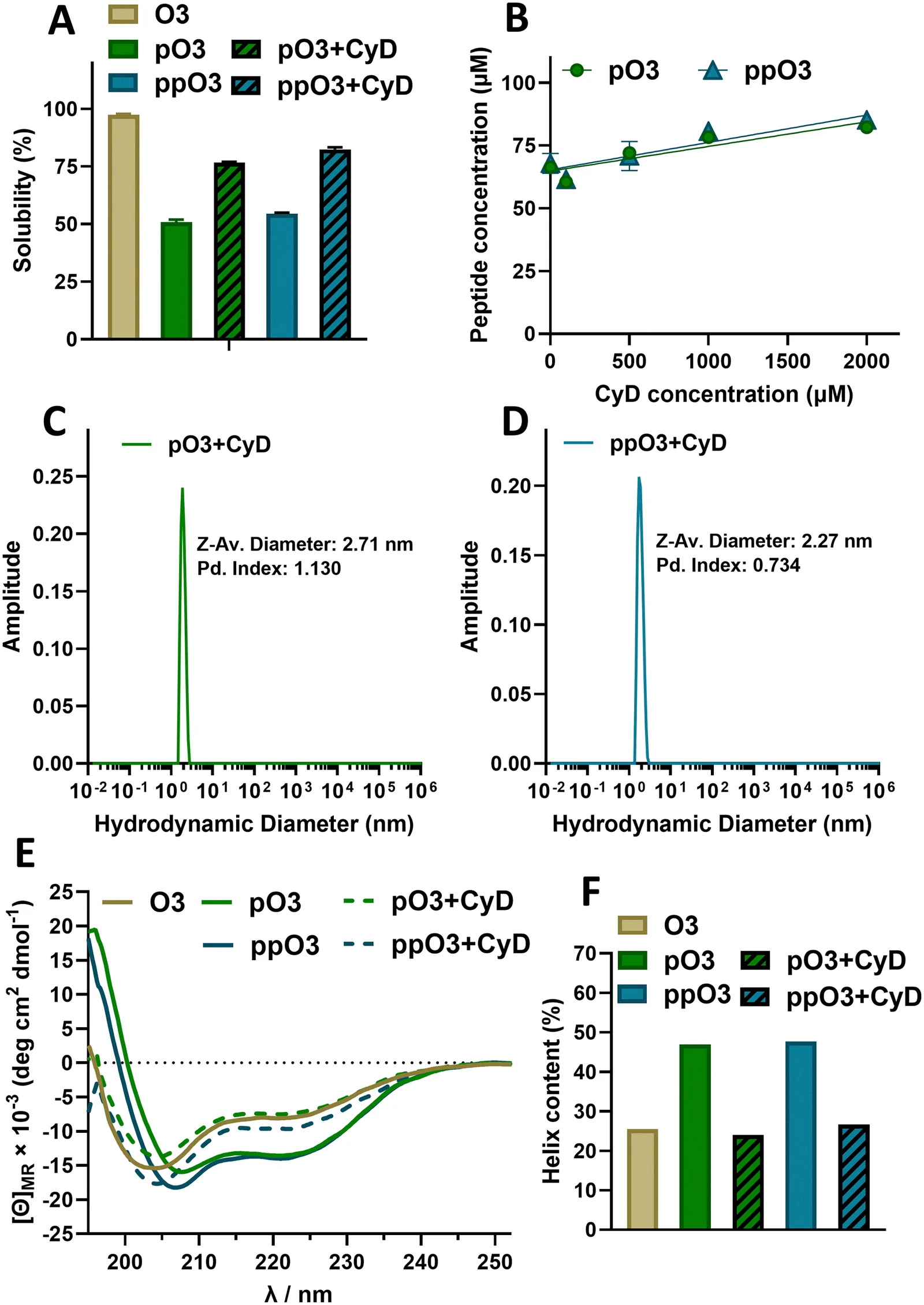
Solubility and structure of the Orai3-derived and complexed peptides. (A) Peptides O3, pO3 and ppO3 were dissolved in PBS (100 µM; pH = 7.4) or in a PBS solution of CyD (2 mM) and after 10 min of agitation and filtration, the peptide concentration was determined, and the percentage of solubility was calculated. Bars represent mean ± SD (*N* = 3). (B) Complexation parameters were calculated from the phase solubility diagram, recorded after 24 hours of agitation, applying various CyD concentrations. Each data point represents the average of three measurements, ± SD. Dynamic light scattering (DLS) measurement of the (C) pO3 + CyD and the (D) ppO3 + CyD complexes was performed using a W130i DLS instrument. Mass distribution of the unfiltered samples was recorded at 20 °C for 10 s, repeated 10 times, averaged and analysed using iSize 3.0 software. (E) Far-UV circular dichroism spectra of the PBS solutions of the peptides at 25 °C were measured on a JASCO J-1500 spectropolarimeter. (F) Helix content of the peptides determined by spectral deconvolution using CDNN software.

The aggregation properties and the interaction between the Orai3-derived lipopeptides and RAMEA cyclodextrin were initially investigated *via* a solubility study, after 10 min of agitation in PBS or in a 2 mM CyD/PBS solution (Fig. 1A), using a 0.22 µm filter. The selected time interval is justified by the clinically feasible reconstitution time established for analogous peptide-based therapeutics, while remaining within the tolerance limits of the peptide substances to agitation-induced mechanical stress. The saturation solubility of the palmitoylated peptide pO3 in PBS was 54.5 µM, while that of the palmitoylated peptide including the PEG spacer (ppO3 peptide) was close to this value at 55.1 µM. It is an interesting observation that the PEG group did not cause a significant increase in solubility, but essentially, this represents a manageable solubility for biological measurements. After the addition of RAMEA cyclodextrin to the former two lipopeptides, their solubility significantly increased (*P* < 0.0001 Student's *t*-test) and reached 76.8 and 82.4%, respectively. The 2 mM CyD concentration used corresponds to 1.8% (w/v), which represents a 1 : 20 molar ratio relative to the lipopeptides. Besides, the resulting saturated solubility of approximately 80 µM is sufficient for the preparation of a stable solution formulation of the lipopeptides.

To study the equilibrium states, the lipopeptides were allowed to interact with CyD for 24 hours, using various CyD concentrations. The solubility of pO3 and ppO3 increased linearly with the CyD concentration, with a slope less than 1, indicating the formation of an inclusion complex with a 1 : 1 stoichiometry.^41^ The apparent stability constants (*K*_(1 : 1)_), determined from the linear regression, using the equation of *K*_(1 : 1)_ = slope/*C*_0_ × (1 − slope), where *C*_0_ is the peptide concentration at µM CyD, were 149.3 M^−1^ (pO3) and 162.2 M^−1^ (ppO3), respectively, indicating a weak interaction between the fatty acid and the CyD molecule (Fig. 1B). The data suggest that the solubilization capacity of CyD is similar for pO3 and ppO3 lipopeptides, and that the PEG linker does not affect the stability of the inclusion complex.

To determine the size distribution of the ppO3 + CyD system, we performed dynamic light scattering (DLS) measurements without filtering the samples. These measurements indicated that a monomeric dispersion had formed with the cyclodextrin, with a typical size ranging from 1 to 3 nm (Fig. 1C and D). This is a typical value for CyD complexes and is due to self-association.^42^ Next, we investigated the stability of the ppO3 peptide and the ppO3 + CyD inclusion complex using RP-HPLC and DLS methods. The results showed that after 24 hours of agitation at 37 °C, the ppO3 peptide remained intact, as evidenced by the RP-HPLC chromatograms. In addition, we observed that the ppO3 + CyD complex also remained stable; in fact, the polydispersity data indicate that the system became more homogeneous after 24 hours of agitation at 37 °C. This conclusion can be drawn from the PDI (polydispersity index) values, which decreased from 0.734 to 0.018 (Fig. S8).

### 2.3. Palmitoylation promotes folding, while the addition of CyD restores the original Orai3 structure

ECD spectroscopic measurements of the PBS solutions of the peptides were performed in the far-UV region (Fig. 1E). The parental O3 peptide has a highly dynamic secondary structure. The curves clearly show that the conjugation of the palmitic acid and palmitic acid/octaethylene glycol modifications causes structural changes in both the pO3 and ppO3 peptide derivatives. These differences suggest an increase in helical secondary structural elements within the peptides (Fig. 1E).

However, when 2 mM CyD is added to the PBS solution, the structure of the pO3 and ppO3 peptides resembles that of O3 in PBS. It appears that the structural stability induced by palmitic acid and PEG8 is altered by cyclodextrins, resulting in a spatial structure similar to that of O3 without linkers. This is supported by the change in the α-helical contribution among the curve-fitting values obtained using CDNN software (Fig. 1F). Similar observations were reported by Lainé *et al.*; the non-complexed lipopeptides exhibited a higher α-helical content and TEM imaging demonstrated an increased propensity for self-assembly into ordered fibrillar aggregates than the cyclodextrin-complexed form.^43^ This increased helicity of the palmitoylated form likely reflects a thermodynamic trade-off in which the entropic cost of conformational ordering is compensated by favourable intermolecular hydrophobic interactions and desolvation, stabilizing backbone hydrogen bonding. This is consistent with the behaviour of weak protein–protein interactions such as STIM1–Orai1, which is strongly influenced by local concentration, molecular crowding and binding kinetics.^44^ In this framework, the cyclodextrin complex may act as a delivery scaffold that promotes membrane incorporation while limiting premature self-association, allowing the peptide to reach the conformational state required for active STIM1 engagement only after reaching the crowded cytosolic environment.

### 2.4. The structural characteristics underlying the peptide assembly

**Fig. 2:**
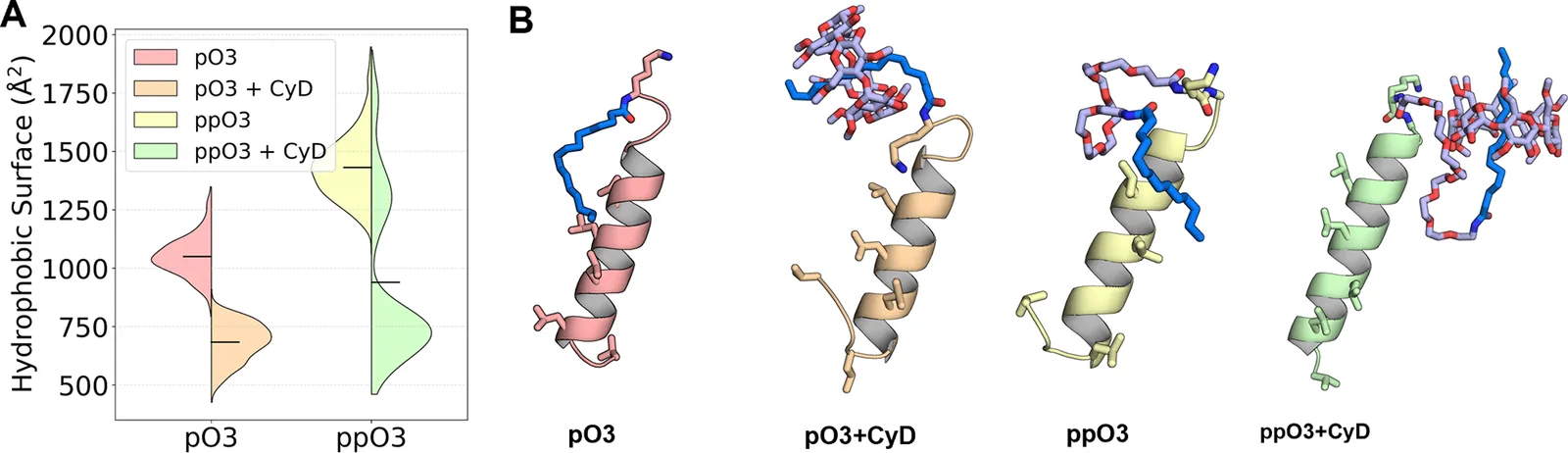
Hydrophobic exposure and representative conformations. (A) Violin plots of the hydrophobic SASA (A^2^) for pO3 and ppO3 with and without CyD. Mean values are indicated by horizontal lines. (B) Representative structures of the studied peptides from the major conformational cluster along the MD simulation. O3 peptide is shown as a cartoon, the palmitoyl moiety (C-atoms in marine blue), CyD (C-atoms in light blue) and the PEG spacer (C-atoms in light blue) are shown as sticks.

To better understand the measured solubility and structural results, we performed 1 µs molecular dynamics (MD) simulations of pO3 and ppO3 systems in aqueous solution, in the presence and absence of CyD, addressing both peptide structuration and palmitoyl solvent exposure. In the absence of CyD, both pO3 and ppO3 exhibit high hydrophobic solvent-accessible surface area (SASA), indicating substantial exposure of the palmitoyl group and therefore their propensity for aggregation (Fig. 2). This effect is more pronounced for ppO3 (1432 ± 135 Å^2^) than for pO3 (1051 ± 86 Å^2^), reflecting greater exposure of the hydrophobic regions in the former system. CyD complexation significantly reduces the hydrophobic SASA in both systems, to 684 ± 84 Å^2^ for pO3 and 940 ± 350 Å^2^ for ppO3. This decrease is consistent with stable encapsulation of the palmitoyl chain within the cyclodextrin cavity, effectively shielding the hydrophobic group from the solvent.

In fact, the PEG chain is also capable of penetrating into the interior of the CyD ring, and M. Kamachi *et al.* have termed this supramolecular assembly a ‘molecular necklace’.^45^ For the peptides we studied, we observe that the main partner of CyD is the palmitoyl chain, and this palmitoyl group either participates in intermolecular interactions or interacts intramolecularly with CyD. Besides, the incorporation of the PEG8 chain further stabilizes the inclusion complex, which is supported by the fact that a lower polydispersity index (PDI) was observed for ppO3 (0.734) than for pO3 (1.130) during the DLS measurements (Fig. 1C and D).

### 2.5. Palmitoylation and CyD formulations modulate Orai3 peptide subcellular localisation

The subcellular localisation and uptake of O3-derived peptides in HEK293 cells were studied using confocal fluorescence microscopy and trypan blue quenching. For confocal microscopy, HEK293 cells were transiently transfected with genetically encoded fluorescent markers for the plasma membrane (mApple-Farnesyl), and endosomes (mCherry-Rab5 and Rab7), followed by incubation with Cf-labelled peptides.

**Fig. 3:**
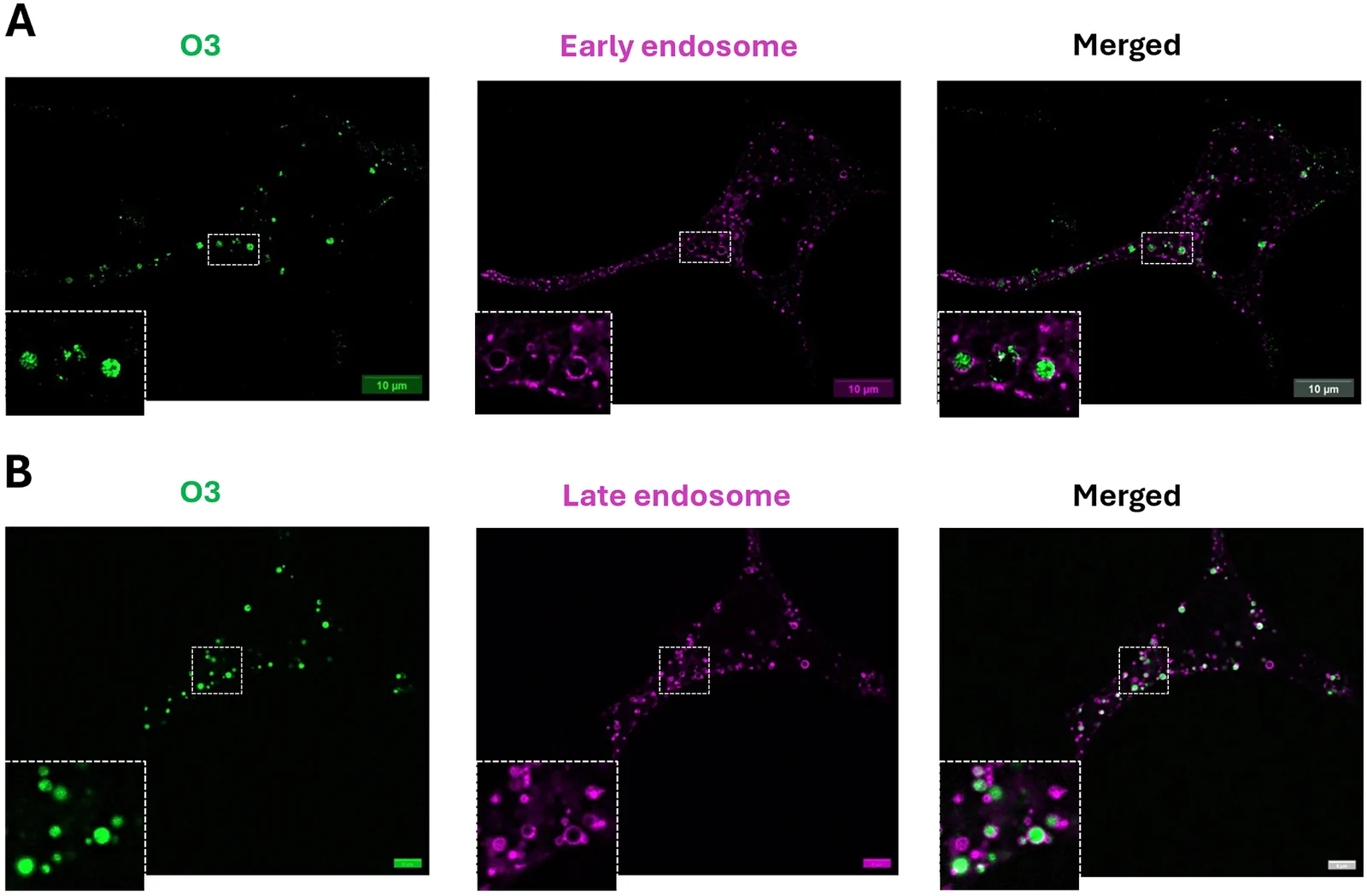
Orai3 peptide localises to Rab5-positive early endosomes and Rab7-positive late endosomes in HEK293 cells. Representative confocal fluorescence microscopy images of HEK293 cells overexpressing the early endosomal marker mCherry-Rab5 or the late endosomal marker mCherry-Rab7 after 20 min of treatment with 10 µM Cf-O3 peptide. Merged images demonstrate colocalization of the peptide with Rab5- and Rab7-positive vesicles. (A) mCherry-Rab5 and (B) mCherry-Rab7. The scale bars are 10 µm (A) and 5 µm (B), which apply to the main images. The insets are shown at 2.7× magnification.

Fluorescence microscopy revealed that O3 displayed predominantly an intracellular, punctate distribution and showed strong localisation in Rab5-positive early endosomes (Fig. 3A), consistent with endocytic uptake. Moreover, O3 was also detected in Rab7-positive degradative late endosomes (Fig. 3B), suggesting trafficking toward the endolysosomal pathway.

**Fig. 4:**
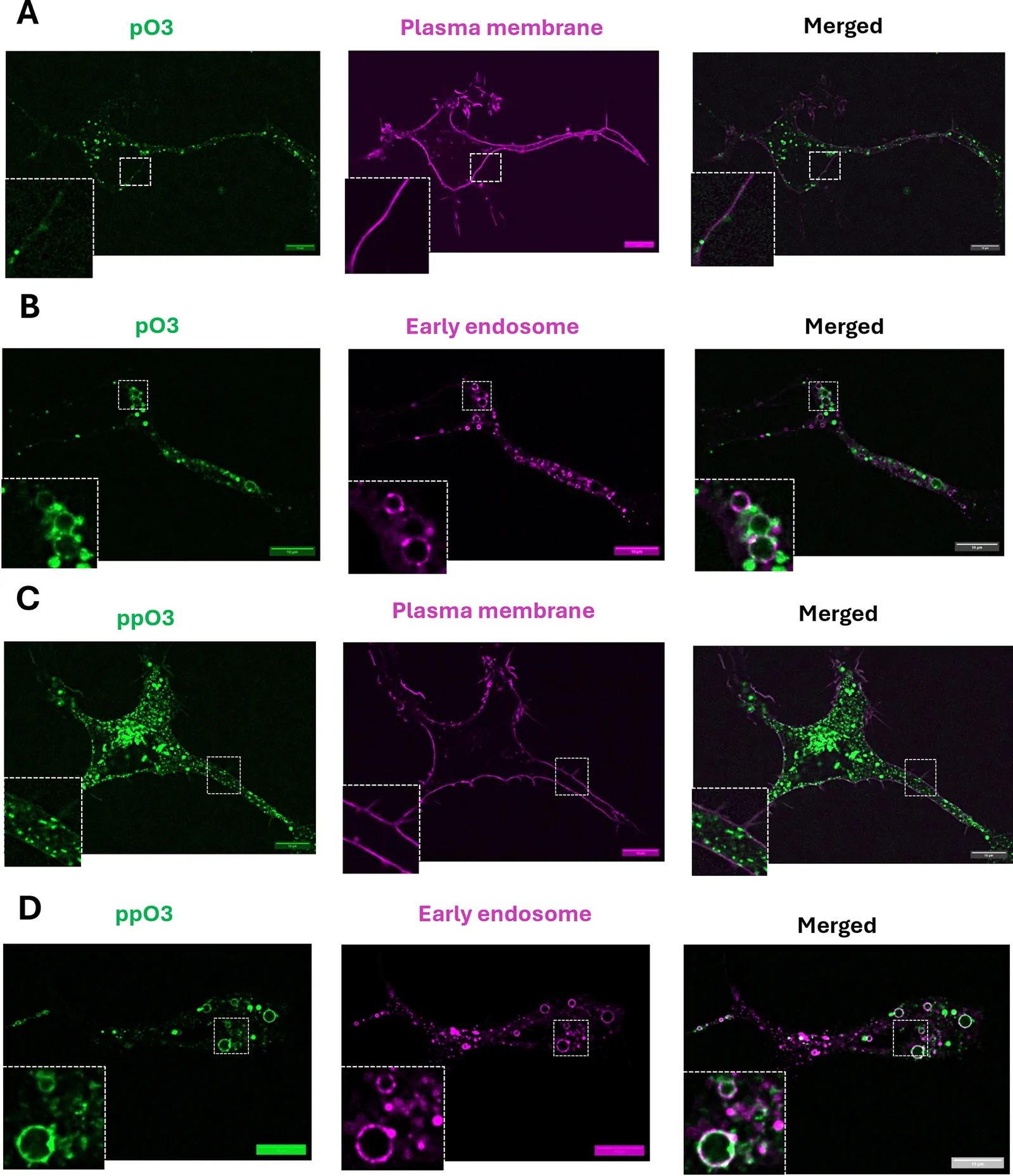
pO3 and ppO3 peptides exhibit multiple localisation patterns in HEK293 cells. Representative confocal fluorescence microscopy images of HEK293 cells overexpressing the plasma membrane marker mApple-Farnesyl or the early endosomal marker mCherry-Rab5 after 20 min of treatment with 10 µM p(Cf)O3 or pp(Cf)O3 peptide. Merged images demonstrate colocalization of the peptides with the plasma membrane marker and Rab5-positive vesicles. (A) p(Cf)O3 + mApple-Farnesyl; (B) p(Cf)O3 + mCherry-Rab5; (C) pp(Cf)O3 + mApple-Farnesyl; (D) pp(Cf)O3 + mCherry-Rab5. Scale bar: 10 µm, which applies to the main images. The insets are shown at 2.7× magnification.

In contrast, both pO3 and ppO3 exhibited heterogeneous subcellular distribution. A minor fraction of each peptide colocalized with the plasma membrane marker (Fig. 4A and C), whereas the majority of the signal was associated with the Rab5 early endosomal membrane (Fig. 4B and D). This indicates concurrent membrane association and fast endocytic uptake. In addition to vesicular fluorescence, a weak diffuse intracellular signal was also observed, consistent with partial cytosolic release from endosomal compartments (Fig. 4C). Previous studies have demonstrated that cell-penetrating peptides can bind to the plasma membrane, undergo aggregation, and form peptide-enriched lipid domains that, upon reaching a critical size, bud off as vesicles.^46,47^ Such processes have been associated with membrane destabilisation and vesicle disruption.^32,33^ A similar mechanism may contribute to the vesicular structures and the diffuse intracellular fluorescence observed here. Notably, ppO3 exhibited higher overall fluorescence intensity than pO3 (Fig. 4C and A), consistent with increased membrane association and cellular uptake. Together, these results indicate that palmitoylation enhances membrane association and promotes endocytic uptake. Incorporation of the PEG8 linker on ppO3 further increased uptake efficiency, leading to the formation of vesicular structures with early endosomal characteristics.

**Fig. 5:**
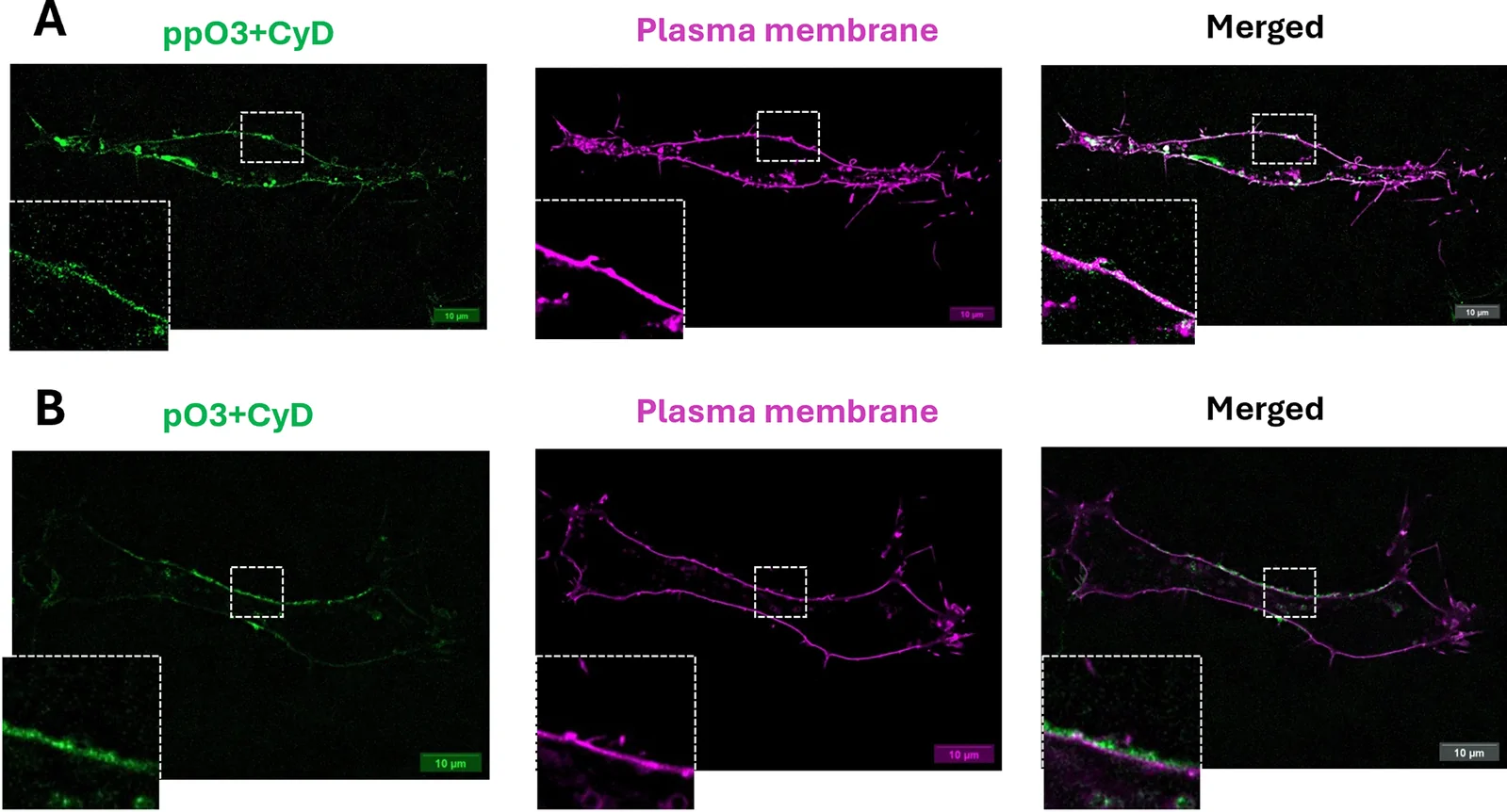
pO3 and ppO3 peptides complexed with CyD localize to the plasma membrane in HEK293 cells. Representative confocal fluorescence microscopy images of HEK293 cells overexpressing the plasma membrane marker mApple-Farnesyl after 20 min of treatment with 10 µM p(Cf)O3 or pp(Cf)O3 peptide complexed with RAMEA cyclodextrin. Merged images demonstrate colocalization of the peptides with the plasma membrane marker. (A) p(Cf)O3 and (B) pp(Cf)O3. Scale bar: 10 µm, which applies to the main images. The insets are shown at 2.7× magnification.

Upon complex formation with CyD, both pO3 and ppO3 were predominantly localized at the plasma membrane, showing strong overlap with the mApple-Farnesyl plasma membrane marker (Fig. 5A and B) and reduced endosomal uptake.

**Fig. 6:**
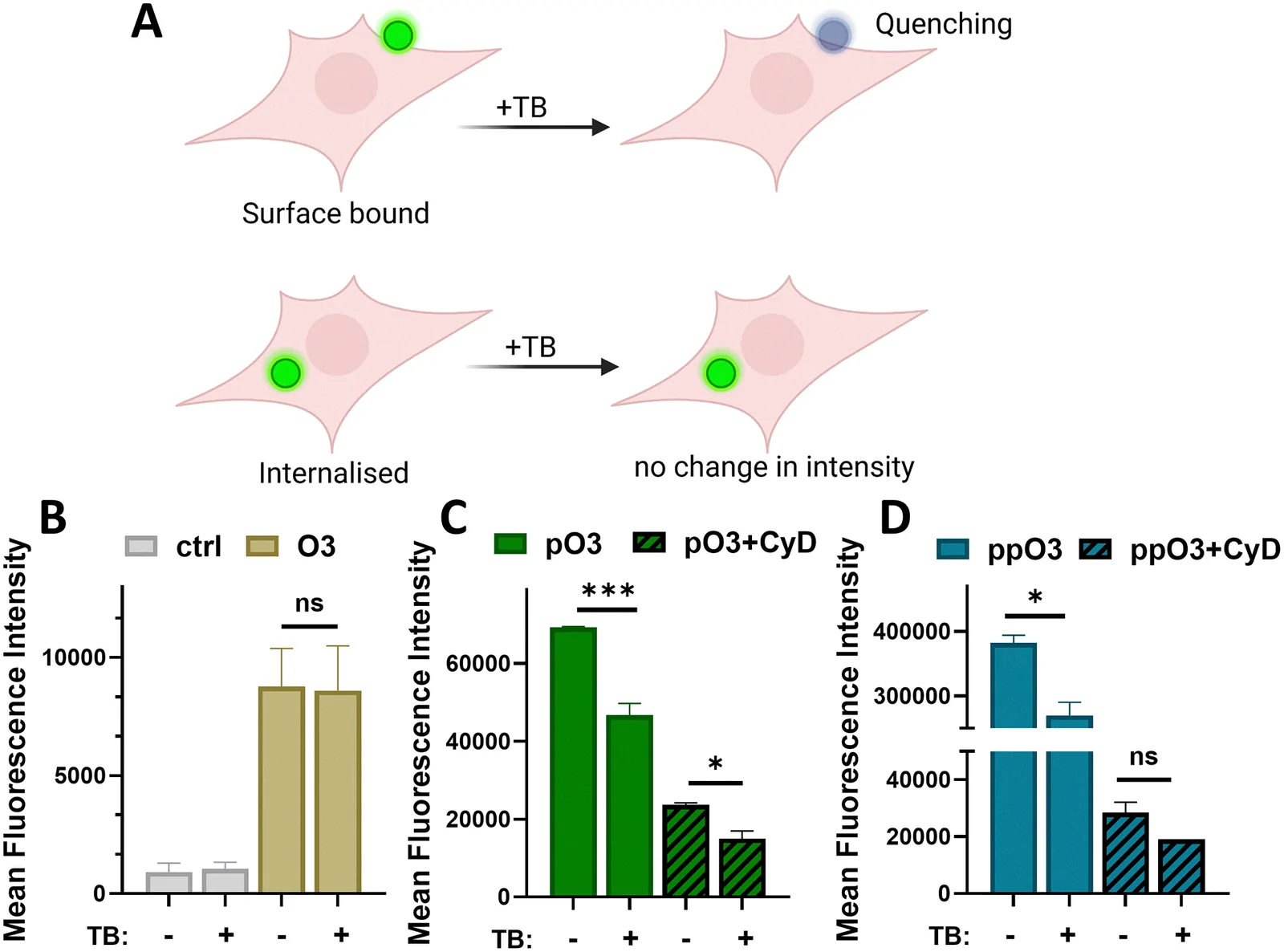
Trypan blue (TB) quenching reveals differential extracellular accessibility of Orai3-derived peptides. (A) Schematic representation of the trypan blue quenching assay. Extracellularly exposed, the surface-bound Cf-labelled peptide is quenched by TB, whereas the internalized or otherwise TB-inaccessible peptide remains fluorescent. (B)–(D) Flow cytometry experiments performed in triplicate; bars represent mean MFI ± SD, *N* = 3. Statistical analysis was performed using Student's *t*-test. Statistical significance is denoted as *P < 0.05, **P < 0.01, and ***P < 0.001. The presence or absence of trypan blue is indicated by + and −, respectively. Peptide variants are indicated by colour: O3 (beige, B), pO3 (green, C), and ppO3 (blue, D). Hatched bars indicate cyclodextrin-complexed peptides.

To distinguish between membrane-associated and internalised peptide populations, cells were incubated with Cf-labelled peptides and then treated with trypan blue prior to flow cytometric analysis. As a membrane-impermeable quencher, trypan blue selectively suppresses fluorescence derived from the extracellular environment, whereas intracellular or otherwise trypan blue-inaccessible fluorescence is preserved^48^ (Fig. 6A).

Flow cytometry analysis revealed no significant change in the mean fluorescence intensity of O3-treated cells following trypan blue treatment, indicating a predominantly intracellular, trypan blue-inaccessible localisation (Fig. 6B). This finding is consistent with the fluorescence microscopy data (Fig. 3A and B). In contrast, both pO3- and ppO3-treated cells showed an approximately 30% reduction in fluorescence intensity upon quenching (Fig. 6C and D). This indicates that a substantial fraction of each non-complexed peptide remained accessible from the extracellular side of the plasma membrane.

CyD complexation reduced the trypan blue quenching for both lipopeptides. For CyD-complexed pO3, a significant quenching effect was observed, indicating that part of the peptide population was still extracellularly accessible (Fig. 6C). For CyD-complexed ppO3, trypan blue treatment caused no statistically significant decrease in fluorescence intensity, suggesting that the Cf fluorophore was largely protected from the extracellular environment under these conditions (Fig. 6D). Together with the predominant plasma membrane-associated signal observed by confocal microscopy, these data indicate reduced extracellular accessibility of the CyD-complexed lipopeptides, particularly ppO3.

This protected fluorescence may reflect deep membrane insertion, steric shielding of the fluorophore, partial redistribution toward the inner leaflet, or a combination of these processes. Trypan blue quenching alone cannot discriminate between these possibilities because the assay reports extracellular fluorophore accessibility rather than membrane sidedness.

Previous FRET-based studies demonstrated that similar lipopeptides can undergo transbilayer translocation and localize at the inner leaflet of the plasma membrane.^49^ However, direct sidedness measurements would be required to confirm this behaviour for the present Orai3-derived lipopeptides.

### 2.6. CyD-complexed ppO3 reduces SOCE in a PEG8-dependent manner

Given the distinct uptake and membrane-localization patterns observed for the different Orai3-derived peptides, we next tested whether these differences translated into functional inhibition of SOCE. Previous studies showed that Orai3 C-terminal fragments inhibit SOCE when anchored to the cytosolic face of the plasma membrane in overexpression systems. Under these conditions, the Orai3-derived sequence peptide interferes with STIM1-dependant activation of Orai channels.^25^ Similar findings obtained with alternative plasma membrane–targeted constructs support the concept that membrane presentation of the Orai3 C-terminus can modulate SOC channel activity.^26^

**Fig. 7:**
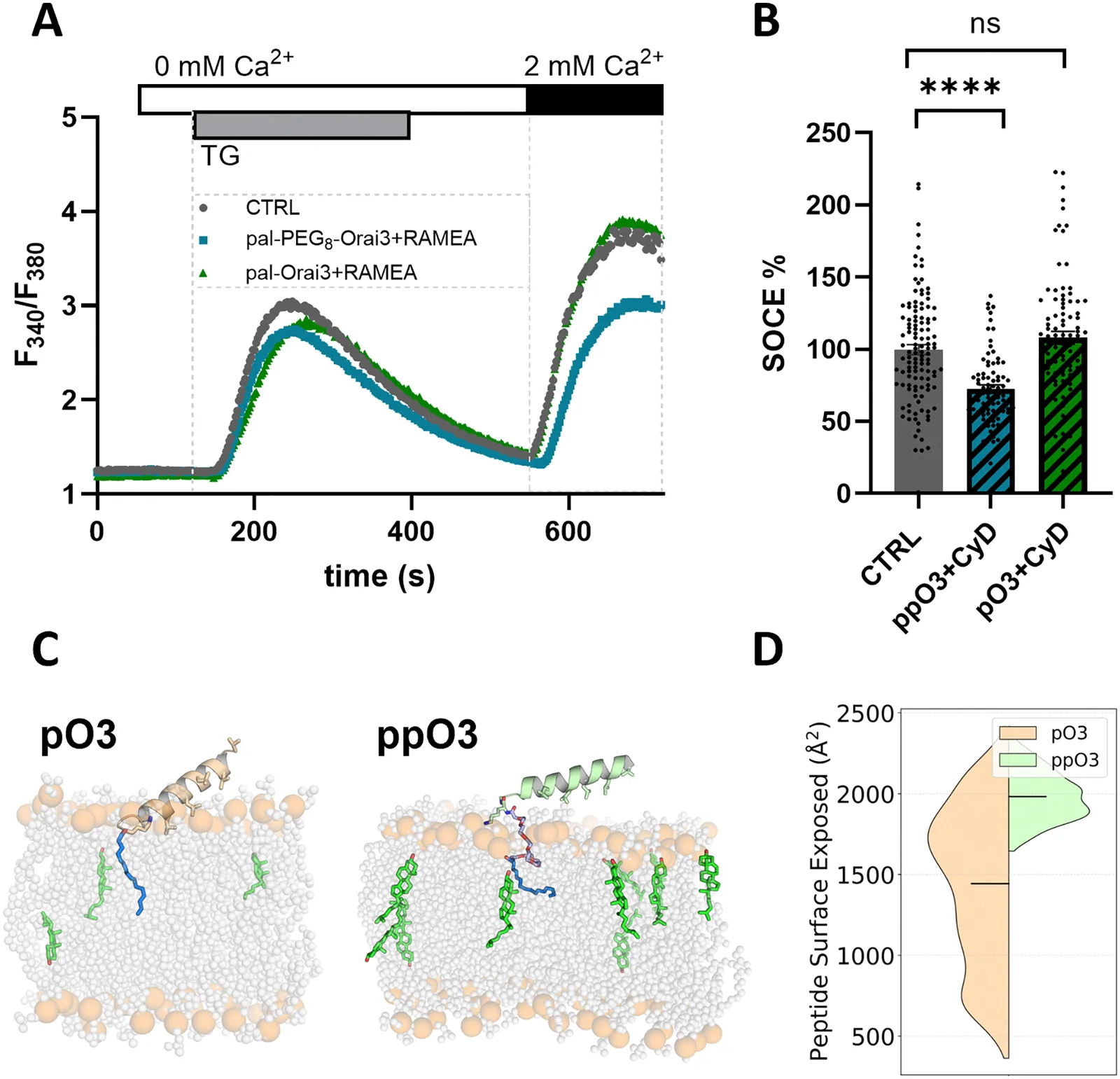
CyD-complexed ppO3 reduces SOCE in a PEG8-dependent manner: (A) Time course of cytosolic calcium transients in HEK293 cells pretreated with CyD, pO3 + CyD, or ppO3 + CyD. (B) SOCE amplitude showing that ppO3 + CyD significantly reduces (*P* < 0.0001 Student's *t*-test) calcium entry compared with the CyD treated control, whereas pO3 + CyD shows no detectable inhibitory effect. Data are presented as mean ± SEM; each dot represents one analyzed cell pooled from three independent cell preparations. CyD control: *n* = 89 cells; pO3 + CyD: *n* = 81 cells; ppO3 + CyD: *n* = 91 cells; *N* = 3 independent cell preparations per condition. Statistical significance was assessed using Student's *t*-test. (C) Representative molecular dynamics simulation snapshot showing increased flexibility and solvent exposure of ppO3 relative to pO3 due to the PEG8 linker. (D) Distribution of the solvent-exposed peptide surface area for pO3 and ppO3 along the MD simulation trajectory.

We therefore measured cytosolic calcium responses in peptide-pretreated HEK293 cells loaded with Fura-2AM using a standard thapsigargin-based protocol. ER stores were depleted with thapsigargin in nominally calcium-free solution, and SOCE amplitude was quantified after calcium readdition. Pretreatment with CyD-complexed ppO3 significantly reduced (*P* < 0.0001 Student's *t*-test) the SOCE amplitude by approximately one-third compared to the CyD-treated control (Fig. 7A and B). In contrast, CyD-complexed pO3 had no detectable effect, with the SOCE amplitude remaining close to control values (Fig. 7B).

The lack of inhibition by pO3 + CyD is informative, since both pO3 and ppO3 showed PM-associated fluorescence under CyD complexed conditions (Fig. 5A and B). Thus, plasma membrane recruitment by palmitoylation alone is not sufficient for functional inhibition. PEG8 appears to be required for a target-accessible presentation of the Orai3-derived sequence. We propose that PEG8 increases the effective distance and conformational freedom between the palmitoyl anchor and the peptide moiety, thereby improving peptide exposure at the membrane interface.

As discussed above, the trypan blue experiments indicate reduced extracellular accessibility of CyD-complexed ppO3, but they do not directly demonstrate complete inner leaflet localization or direct STIM1 binding. Therefore, the SOCE inhibition observed here is best interpreted as a functional effect consistent with improved membrane-associated presentation and possible partial access to the cytosolic STIM1–Orai signaling interface.

Further experiments support this interpretation: non-lipidated O3, which localized predominantly to endosomal compartments, did not affect the SOCE amplitude (Fig. S10A). Similarly, CyD-free ppO3 did not significantly alter SOCE compared with untreated controls (Fig. S10B and S10C), consistent with its stronger endosomal uptake and the presence of a trypan blue-accessible peptide fraction under these conditions (Fig. 4 and 6). The approximately one-third reduction in SOCE amplitude indicates significant but incomplete inhibition. This suggests that CyD complexation and PEG8 incorporation improve functional delivery, but do not convert the entire lipopeptide population into a target-accessible state. A fraction of the peptide may remain extracellularly exposed, retained in inaccessible membrane-associated conformations, or internalized through endocytic pathways. This partial efficiency is also consistent with the lower inhibition observed for the synthetic CyD + ppO3 formulation compared with genetically encoded Orai3 C-terminal constructs directly targeted to the inner leaflet.^26^ Further experiments directly probing peptide sidedness, STIM1 binding, or localization at ER–PM junctions would be required to define the precise molecular mechanism.

### 2.7. MD simulations reveal differential membrane exposure and conformational flexibility of pO3 and ppO3

To better understand why ppO3 is more effective than pO3 in inhibiting SOCE despite both peptides being membrane-anchored, we performed molecular dynamics simulations in which the palmitoyl group of each peptide was embedded in one leaflet of a model lipid bilayer (Fig. 7C). Both pO3 and ppO3 stably remained embedded in the membrane over the course of the simulations, consistent with the palmitoyl-driven membrane association observed experimentally. However, ppO3 adopted more solvent-exposed and flexible conformations at the membrane interface, a behaviour facilitated by the PEG8 linker that decouples the peptide backbone from tight lipid packing. This is reflected in a higher solvent-accessible surface area for ppO3 (1983 ± 150 Å^2^) compared with pO3 (1443 ± 476 Å^2^), as shown in Fig. 7D.

Molecular dynamics simulations show that, in the membrane associated pO3, the peptide N-terminus remains partially buried within the lipid bilayer (Fig. 7C). This is shown by a broad distribution of the solvent-exposed peptide surface area over the simulation trajectory, with values extending to as low as ∼500 Å^2^. In contrast, the PEG8-linked ppO3 peptide exhibited a narrower distribution around ∼2000 Å^2^, indicating higher and more uniform solvent exposure.

### 2.8. Dual role of the PEG8 linker in cellular uptake and target presentation

Incorporation of the PEG8 linker strongly affected the cellular and functional properties of the lipopeptide. The PEG8-containing ppO3 showed an approximately 6-fold higher mean fluorescence intensity than the pO3, indicating clearly enhanced cellular uptake (Fig. 6C and D). Enhanced uptake of PEGylated lipopeptides has previously been associated with increased hydrophilicity, improved membrane accessibility and reduced aggregation. Surprisingly, incorporation of the PEG8 linker did not increase the solubility and aggregation propensity of ppO3 compared to pO3 (Fig. 1A). In addition to promoting uptake, PEG linkers act as spacers that improve target presentation. By separating the lipid anchor from the peptide sequence, PEG reduces steric hindrance and increases conformational flexibility. Linker length further influences membrane interaction, insertion kinetics, peptide accessibility and target engagement. Intermediate linker lengths (PEG6-12) have been reported to provide the optimal balance between membrane insertion and dynamic peptide presentation.^50^ Consistent with these observations, our MD simulations of pO3 and ppO3 in the membrane further support that PEG8 enhances peptide conformational flexibility and presentation at the membrane interface. Collectively, these findings support the dual role of the PEG8 linker in enhancing cellular uptake and optimizing target presentation.

## 3 Experimental

### 3.1. Peptide synthesis and characterisation

Peptides were prepared by continuous flow peptide synthesis on a commercially available equipment (HPPS-4000, METALON Ltd, Hungary) using elevated temperature (*T* = 80 °C) and elevated pressure (*p* = 60–80 bar), applying a method further developed by us.^38–40^ The system is based on a standard Jasco LC-4000 series HPLC system, equipped with an autoinjector and a backpressure regulator. Fmoc-amino acids (purchased from Iris Biotech GmbH, Marktredwitz, Germany) were dissolved in *N*-methylpyrrolidone (NMP) and measured into a vial with an equivalent amount of OxymaPure (ethyl-2-cyano-2-(hydroxyimino)acetate). *N*,*N*′-diisopropylcarbodiimide (DIC) reagent was added using the autoinjector, and the activated amino acid solution was injected into the system, where it flowed through a PEEK fixed bed column, filled with TentaGel S RAM resin (capacity = 0.24 mmol g^−1^). For Fmoc cleavage, 20 V/V% piperidine/*N*,*N*′-dimethylformamide (DMF) solution was used and monitored by a UV detector. After the completion of the synthesis, the peptide–resin was removed from the column and palmitic acid was coupled manually using DIC/HOBt coupling regents. The PEG linker was introduced as the Fmoc-NH-PEG(8)-COOH reagent (Iris Biotech GmbH), using the same coupling protocol. For the fluorescein labelled analogues, the 5(6)-carboxyfluorescein (Cf) moiety was coupled on a side chain of a lysine residue. For that, orthogonally protected Lys amino acid was used, namely Fmoc-Lys(Dde)-OH (Iris Biotech GmbH). The (4,4-dimethyl-2,6-dioxocyclohex-1-ylidene)ethyl (Dde) protecting group was cleaved with 2% N_2_H_2_/DMF solution, and then the free amino group was reacted with the Cf in the presence of DIC/HOBt coupling reagents. After the completion of the synthesis, peptides were cleaved from the resin using a mixture of 94% trifluoroacetic acid (TFA), 3% triisopropylsilane (TIS), and 3% H_2_O. The crude product was precipitated in cold diethyl ether, centrifuged (4000 rpm, 5 min), and then lyophilized from H_2_O/acetonitrile (AcN).

Reverse phase high-performance liquid chromatography (RP-HPLC) purification was performed on a Shimadzu HPLC System (Kyoto, Japan) using a Phenomenex Jupiter Proteo C12 column (10 µm, 90 Å; 150 × 21.2 mm) with linear gradient elution using 0.1% TFA in H_2_O (eluent A) and 0.1% TFA in AcN : H_2_O = 80 : 20 (v/v) (eluent B). Analytical RP-HPLC chromatograms were recorded on a Jupiter Proteo (4 µm, 90 A; 150 × 4.6 mm) column, or on a ReproShell ODS-1, 5 µm, 250 × 4.6 mm column (Dr Maisch High-Performance LC GmbH, Ammerbuch-Entringen Germany). Using a gradient elution of 5–100 B% in 20 min. The high-resolution mass spectra (HR-MS) of the purified peptides were measured on a Thermo Scientific QExactive Focus Hybrid Quadrupole-Orbitrap Mass Spectrometer (Waltham, MA, USA) and data were analysed using Xcalibur program (Thermo Fisher Scientific).

### 3.2. Saturated solubility and phase solubility studies

Samples were prepared by dissolving the Cf-labelled peptides in PBS or in 2 mM PBS solution of RAMEA CyD (DS ∼ 11, CycloLab Ltd, Budapest, Hungary) at a concentration of 100 µM. The resulting suspensions were agitated at room temperature for 10 minutes, and then filtered through 0.22 µm Nylon centrifuge filters (7.000 rpm). Forty microliters of the filtrates were transferred to a UV-STAR 96-well half area microplate (Greiner Bio-One) and the optical density was measured using a plate reader (MultiSkan Go, Thermo Scientific) at *λ* = 496 nm. Based on a previously recorded calibration curve, the concentration of the samples was defined and the saturated solubility, which was given as the ratio of filtered and non-filtered samples, was calculated.

For the phase solubility studies, CyD solution at increasing concentrations from 0.1 to 2 mM was prepared in PBS, and then the peptides were dissolved at a concentration of 100 µM, and slowly agitated at room temperature for 24 hours. Then, the samples were filtered and measured as described above. A linear regression was applied to the data points obtained, and the apparent stability constants (*K*_(1 : 1)_) was calculated as follows:*K*_(1 : 1)_ = slope/(*C*_0_ × (1 − slope)),where *C*_0_ is the peptide concentration at 0 mM CyD.

### 3.3. Electronic circular dichroism (ECD)

The UV-CD measurements were performed on a JASCO (Tokyo, Japan) J-1500 spectropolarimeter fitted with a Peltier. Each spectrum was the average of five scans collected in the far UV (195–260 nm) range with a 0.1 mm path length quartz cell. The following settings were used throughout the measurements: a temperature control system at room temperature, a bandwidth of 1, a step size of 0.2 nm, a response time of 4 s and a scan rate of 50 nm min^−1^.

All spectra were obtained as the average of five scans and were corrected by subtracting the solvent spectrum acquired under identical conditions. All CD data were processed from millidegrees (mDeg) to mean residue ellipticity (deg cm^2^ dmol^−1^) using the Spectra Analysis function of Jasco Spectra Manager, to account for the concentration differences.

The percentage of the protein secondary structure content in the presence of the G3Si PEG6000 dendrimer was calculated using CDNN circular dichroism spectroscopy deconvolution software version 2.1 (Applied Photophysics Ltd, Surrey, United Kingdom).

### 3.4. Dynamic light scattering (DLS)

Size distribution of the peptide + CyD complexes was measured using a W130i DLS instrument (AvidNano Ltd, High Wycombe, UK) equipped with a diode laser (660 nm) and a photodiode detector. Samples containing 100 µM pO3 or ppO3 peptides + CyD in PBS were measured in a final volume of 80 µl without filtration in low-volume disposable cuvettes with a 1 cm path length (UVette, Eppendorf Austria GmbH). The time-dependent autocorrelation functions were measured for 10 s, repeated 10 times and averaged. Data analysis to determine the average hydrodynamic diameter together with the standard deviation and the polydispersity was performed using iSize 3.0 software.

### 3.5. Cell culture and transfection

Cell culture and transfection procedures were conducted with human embryonic kidney 293 (HEK293) cells. The cells were cultured in Dulbecco's Modified Eagle Medium (DMEM) supplemented with 10% foetal bovine serum (FBS), HEPES (10 mmol L^−1^), l-glutamine (2 mmol L^−1^), streptomycin (100 µg mL^−1^), and penicillin (100 U mL^−1^) at 37 °C and a 5% CO_2_ atmosphere.

For microscopy experiments requiring transfection, the culture medium was aspirated, and HEK293 cells were seeded in 35 mm dishes and grown until they reached 60–70% confluency. Transfection was carried out using 0.25 µg of plasmid DNA and Lipofectamine 2000 Transfection Agent (Thermo Fisher Scientific, Vienna, Austria), following the manufacturer's instructions. After incubating the cells for 1 day, they were rinsed with PBS, detached using 250 µL of Accutase for 5 minutes at 37 °C, and the resulting cell suspension was centrifuged for 4 minutes at 400 × *g*. Cells were then resuspended in fresh medium and seeded onto coverslips submerged in fresh DMEM medium at a ratio of 3 : 1 and incubated overnight before conducting microscopy experiments. For plasma membrane localization, the cells were transfected with the plasma membrane marker mApple-Farnesyl, available from Addgene (plasmid #54899). Early endosomes were labelled with mCherry-Rab5a-7 (Addgene #55126), and late endosomes were labelled with mCherry-Rab7a-7 (Addgene #55127).

### 3.6. Flow cytometry

Internalization of the Cf-labelled peptides was measured in HEK293 cells. One day before the experiment, the cells were seeded in a 24-well tissue culture plate (Sarstedt; 50 000 cells per well in 0.5 mL 10% FBS containing DMEM). The next day, the medium was removed, and the cells were treated with 10 µM peptides with or without CyD (2 mM) in PBS. After 30 min of incubation, the cells were transferred to FACS tubes, and then washed with PBS. The intracellular fluorescence intensity of the cells was measured with a CytoFLEX flow cytometer (Beckman Coulter, Brea, California, USA) on an FITC fluorescence channel (excitation *λ* = 488 nm; emission at *λ* = 525/40 nm). All measurements were performed in triplicate, and the mean fluorescent intensity values together with SD were presented. Statistical analysis was performed using Student's *t*-test.

### 3.7. Calcium imaging experiments

Intracellular Ca^2+^ dynamics were assessed using Fura-2 ratiometric imaging. Initially, HEK293 cells on coverslips were incubated with 1 µM Fura-2AM for 20 minutes in calcium containing Tyrode buffer comprising (in mM): 138 NaCl, 5 KCl, 2 CaCl_2_, 1 MgCl_2_, 10 glucose, and 10 HEPES adjusted to pH 7.4 using NaOH. Subsequently, the coverslip was washed three times and incubated for 10 minutes in calcium containing Tyrode buffer supplemented with the indicated concentration of synthetic peptides and then placed in a perfusion chamber mounted on an inverted microscope (Olympus IX71; Vienna, Austria) equipped with a 20×/0.75 objective and perfused with designated solutions at room temperature. The ratios were recorded using Live Acquisition v2.6 software (FEI, Planegg, Germany) and the cells were alternatively excited using 340/26 and 387/11 nm filters (Semrock, Rochester, NY, USA) within an Oligochrome excitation system (FEI). Fluorescent images were captured using a 510/84 nm emission filter (Semrock) and an ORCA-03G digital CCD camera (Hamamatsu, Herrsching am Ammersee, Germany). Sequential excitation at wavelengths of 340 nm (F340) and 380 nm (F380) was performed at 2 second intervals, with emission fluorescence collected at 505 nm. Intracellular Ca^2+^ levels are represented by the F340/F380 ratios. The SOCE amplitude was calculated as the increase in the F340/F380 ratio upon calcium readdition after thapsigargin-induced store depletion. The experiments were performed using three independent cell preparations per condition. For Fig. 7, the number of analysed cells for each condition is indicated in the figure legend. Individual cells were plotted to show cell-to-cell variability, and data are presented as mean ± SEM. Statistical analysis was performed using Student's *t*-test. Statistical significance was denoted as ****p < 0.0001.

### 3.8. Confocal microscopy and colocalization experiments

Cells were transfected with different cell compartment markers as explained in Section 3.4 and pretreated with the 10 or 100 µM peptide solutions as indicated in 2 mM Ca^2+^ Tyrode buffer containing (in mM): 2 CaCl_2_, 138 NaCl, 1 MgCl_2_, 5 KCl, 10 HEPES and 10 Glucose. Imaging was performed using a Nikon HCS Ti2 Eclipse (Celesta V2) microscope (Nikon Europe B.V., Amstelveen, The Netherlands) at the Core Facility Bioimaging, Medical University of Graz. Samples were imaged with a 100× oil immersion objective (1.45 NA) and excitation was provided by 488 and 561 nm laser lines. Emission was collected using an MXR10018 1ST 4000 KG Photometrics BSI Express CMOS camera/PMT, with spectral detection adapted to the fluorophores employed. Images were acquired using NIS-Elements C software with JOBS (v5.42.07) and exported in ND2/TIFF/other formats. Acquisition parameters (laser power, detector gain, and scan speed) were optimized to minimize photobleaching and phototoxicity while maintaining a high signal-to-noise ratio.

### 3.9. System preparation and MD simulations

The initial peptide was obtained using AlphaFold2^51^ and parametrized using ff19SB.^52^ The palmitoyl moiety was obtained from Lipid21^53^ and constructed onto the peptide in PyMOL^54^ to generate pO3; the PEGylated construct (ppO3) was constructed analogously and the lipopeptide residues were parametrized using the general AMBER Force Field 2 (GAFF2)^55^ accordingly.^56^ Alpha-cyclodextrin was obtained initially from PDB 4FEM^57^ and methylated following previous research^56^ and parametrized using GLYCAM 06j.^58^ The aliphatic chain of the palmitic acid was docked within the cyclodextrin following previous research.^56^ Once systems were built, *in vacuo* minimization was carried out,^59^ and consecutively embedded in a cubic TIP3P water box and neutralized with K^+^ and Cl^−^ ions, with a buffering distance between solute and box limits of 13 Å. Membrane systems were built using PACKMOL-Memgen software^60^ with a mixed lipid bilayer composed of POPC and cholesterol in a 9 : 1 ratio. For each system, energy minimization was carried out in three successive stages, relaxing first hydrogen atoms, then solvent molecules, and finally the entire system. The minimized systems were then heated from 100 to 300 K over 50 ps under constant volume (NVT) conditions using a Langevin thermostat^61^ with a collision frequency of 1.0 ps^−1^, while harmonic restraints of 40 kcal mol^−1^ Å^−2^ were applied to all solute atoms to preserve the initial geometry during equilibration. The systems were subsequently equilibrated at 300 K under constant pressure (NPT) conditions using a Berendsen barostat.^62^ Long-range electrostatic interactions were treated using the particle mesh Ewald (PME) method,^63^ and a 10 Å cutoff was applied to non-bonded interactions. The SHAKE algorithm^64^ was used to constrain bonds involving hydrogen atoms, and hydrogen mass repartitioning^65^ was applied, enabling a 4 fs integration time step. For each system, heating was performed in three independent replicates with different initial velocities. The resulting equilibrated structures were then used as starting points for three 1 µs production MD simulations, giving a total simulation time of 3 µs per system. Trajectory analyses were carried out using MDAnalysis^66^ and CPPTRAJ.^67^

## 4. Conclusions

Palmitoylation has been identified as a powerful strategy for the membrane targeting of biologically active peptides. However, their therapeutic application is limited due to aggregation and inefficient membrane delivery. In this study, we demonstrate that supramolecular complexation of palmitoylated Orai3-derived peptides with randomly methylated α-cyclodextrin overcomes these limitations and enhances plasma membrane localization and reduces endosomal trapping.

**Fig. 8:**
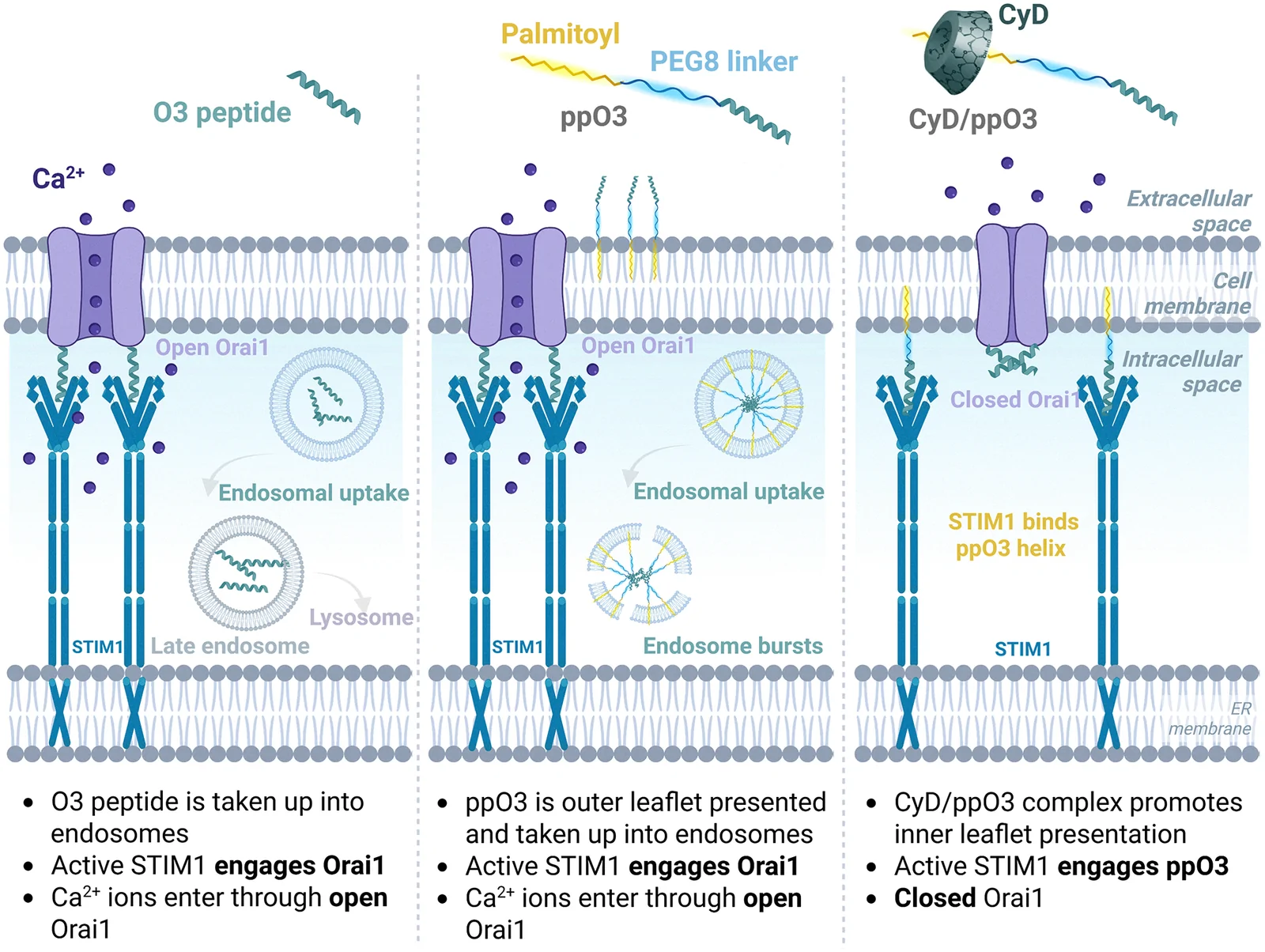
Proposed model of peptide delivery and SOCE modulation. Free O3 peptide is mainly internalized into endosomal compartments and does not inhibit SOCE (left). Palmitoylated ppO3 associates with the plasma membrane but also undergoes endosomal uptake, limiting its access to the cytosolic STIM1–Orai signaling interface (center). CyD complexation reduces extracellular accessibility of ppO3 and favours a protected plasma membrane-associated state. In this model, the PEG8 linker improves peptide exposure at the membrane interface, enabling partial interference with STIM1-dependent Orai activation and reducing Ca^2+^ influx (right).

As illustrated in Fig. 8, inhibition of the SOC channel requires not only membrane anchoring, but also correct membrane presentation of the peptide. While both palmitoylated constructs localized to the plasma membrane, only the PEG8-containing ppO3 peptide inhibited the SOC channel, highlighting the importance of linker-dependent accessibility and target engagement. Molecular dynamics simulations together with cellular quenching experiments suggest that the PEG8 linker improves peptide flexibility and inner leaflet accessibility, thereby facilitating productive interaction with STIM1. Overall, this work highlights the importance of controlling lipopeptide organization, membrane orientation, and target accessibility for the modulation of membrane-associated protein–protein interactions. These findings provide a general design strategy for the development of membrane-targeted peptide therapeutics with improved intracellular activity.

## Author contributions

J. T. M., B. D. G., S. C. and V. F.: investigation, visualization, methodology, formal analysis, and writing – original draft. P. A. S. M. and R. S.: investigation and writing – review & editing. B. B. and K. H.: conceptualization, investigation, supervision, validation, funding acquisition, project administration, writing – original draft, and writing – review & editing. All authors read and approved the final manuscript.

## Conflicts of interest

There are no conflicts of interest to declare.

## Supplementary Material

MA-007-D6MA00756B-s001

## Data Availability

The data supporting this article have been included as part of the supplementary information (SI), Supplementary information: contains the analytical characterization of the peptides, the stability and size distribution of the ppO3 + CyD system, as well as the results of toxicity studies and control calcium measurements of O3-peptides and derivatives. See DOI: https://doi.org/10.1039/d6ma00756b.
