# Supplementary material for: A supramolecular complex of palmitoylated Orai C-terminal peptide and cyclodextrin targets the plasma membrane to reduce store-operated calcium channel activity
Source: Mater Adv. 2026 Aug 21;7(18):9070–81. doi: 10.1039/d6ma00756b (PMC13495069; doi:10.1039/d6ma00756b)
Supplement: Supplementary file 1 [file MA-007-D6MA00756B-s001.pdf]

Supplementary Information (SI) for Materials Advances.
This journal is © The Royal Society of Chemistry 2026

Supplementary Information

Supramolecular complex of palmitoylated Orai C-terminal peptide and cyclodextrin targets plasma membrane to reduce store-operated calcium channel activity

Juan Toledo-Marcos,[a] Bruno Di Geronimo,[b] Sanja Curcic,[a] Viktor Farkas,[c] Pedro A Sánchez-Murcia,[df] Rainer Schindl,[af] Bernadett Bacsa,*[af] and Kata Horváti*[e]

[a] Division of Medical Physics and Biophysics, Gottfried Schatz Research Center, Medical University of Graz, Neue Stiftingtalstrasse 6, 8010 Graz, Austria
[b] School of Chemistry and Biochemistry, Georgia Institute of Technology, 901 Atlantic Drive NW, Atlanta, Georgia 30332-0400.
[c] HUN REN ELTE Protein Modeling Research Group, Eötvös Lorand University, Budapest, Hungary
[d] Laboratory of Computer-Aided Molecular Design, Division of Medicinal Chemistry, Otto-Loewi Research Center, Medical University of Graz, Graz (Austria)
[e] MTA–HUN-REN TTK Lendület "Momentum" Peptide-Based Vaccines Research Group, Institute of Materials and Environmental Chemistry, HUN-REN Research Centre for Natural Sciences, H-1117 Budapest, Magyar Tudósok Krt. 2, Hungary
[f] BioTechMed-Graz, Austria, Auenbruggerplatz 30, 8036 Graz, Austria
[g] *Corresponding authors, equal contribution

Table of Contents:

Figure S1-S6. High-Resolution Mass Spectrometric Analysis of the Peptides

Figure S7. RP-HPLC Analysis of the Peptides

Figure S8. Stability and size distribution of the ppO3+CyD system.

Figure S9. *In vitro* Toxicity of the Compounds.

Figure S10. Calcium Measurements of O3 and ppO3 in HEK293 Cells

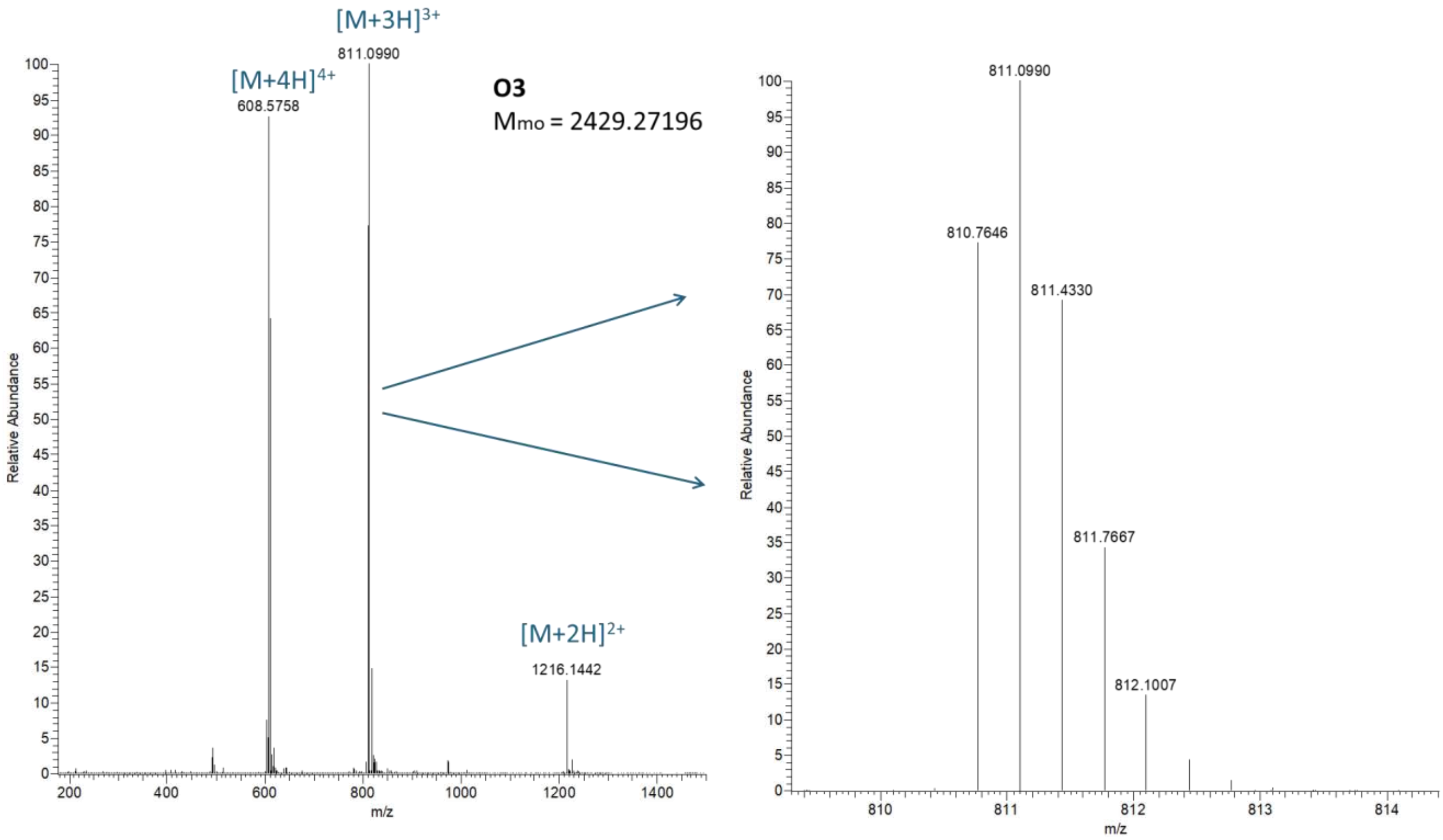

Fig. S1. High-resolution mass spectrum, measured on a Thermo Scientific Q Exactive Focus Hybrid Quadrupole-Orbitrap Mass Spectrometer (Thermo Fisher Scientific Inc.). Full *m/z* range and the magnification of the peak corresponding to the $[M+3H]^{3+}$ ion.

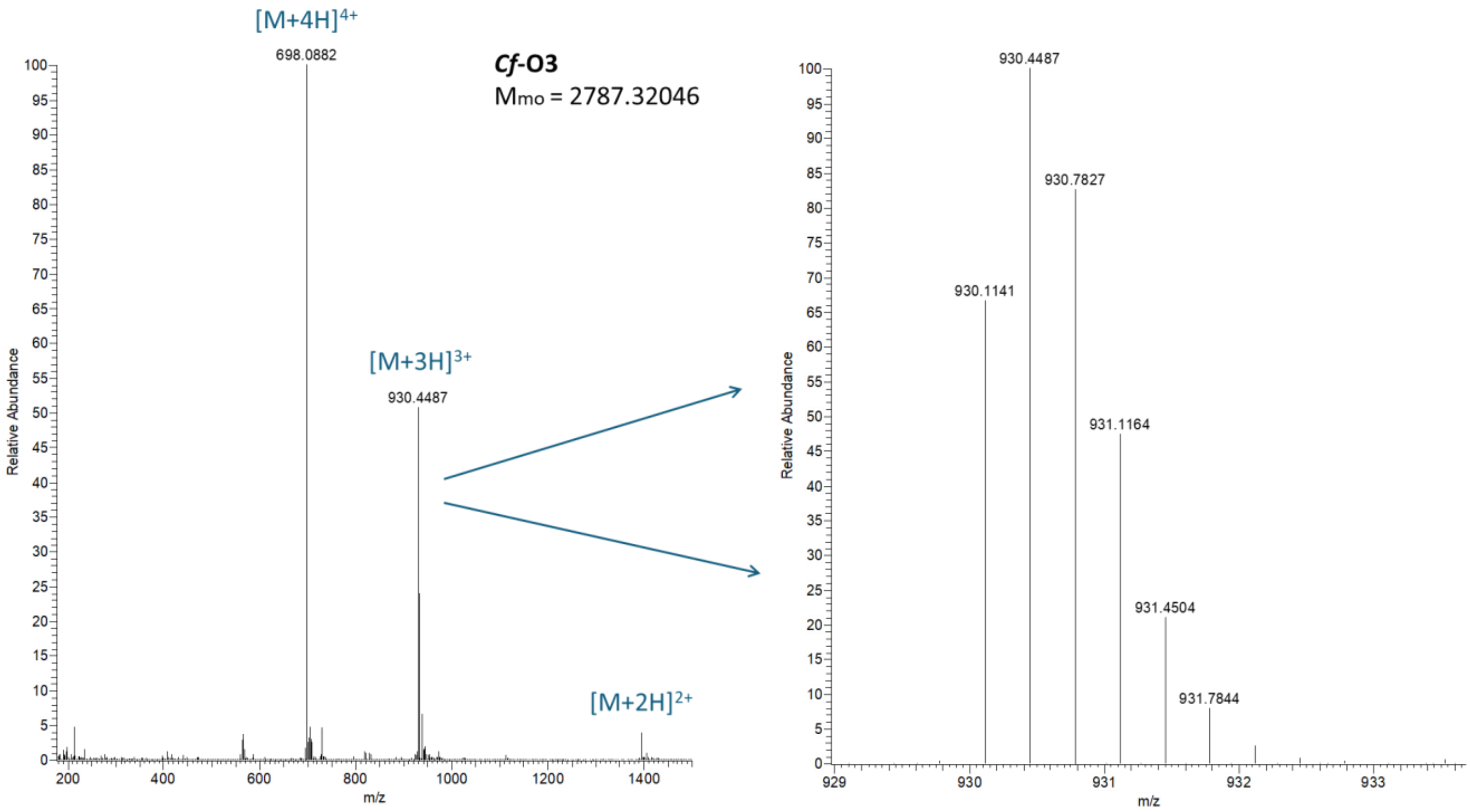

Fig. S2. High-resolution mass spectrum, measured on a Thermo Scientific Q Exactive Focus Hybrid Quadrupole-Orbitrap Mass Spectrometer (Thermo Fisher Scientific Inc.). Full *m/z* range and the magnification of the peak corresponding to the $[M+3H]^{3+}$ ion.

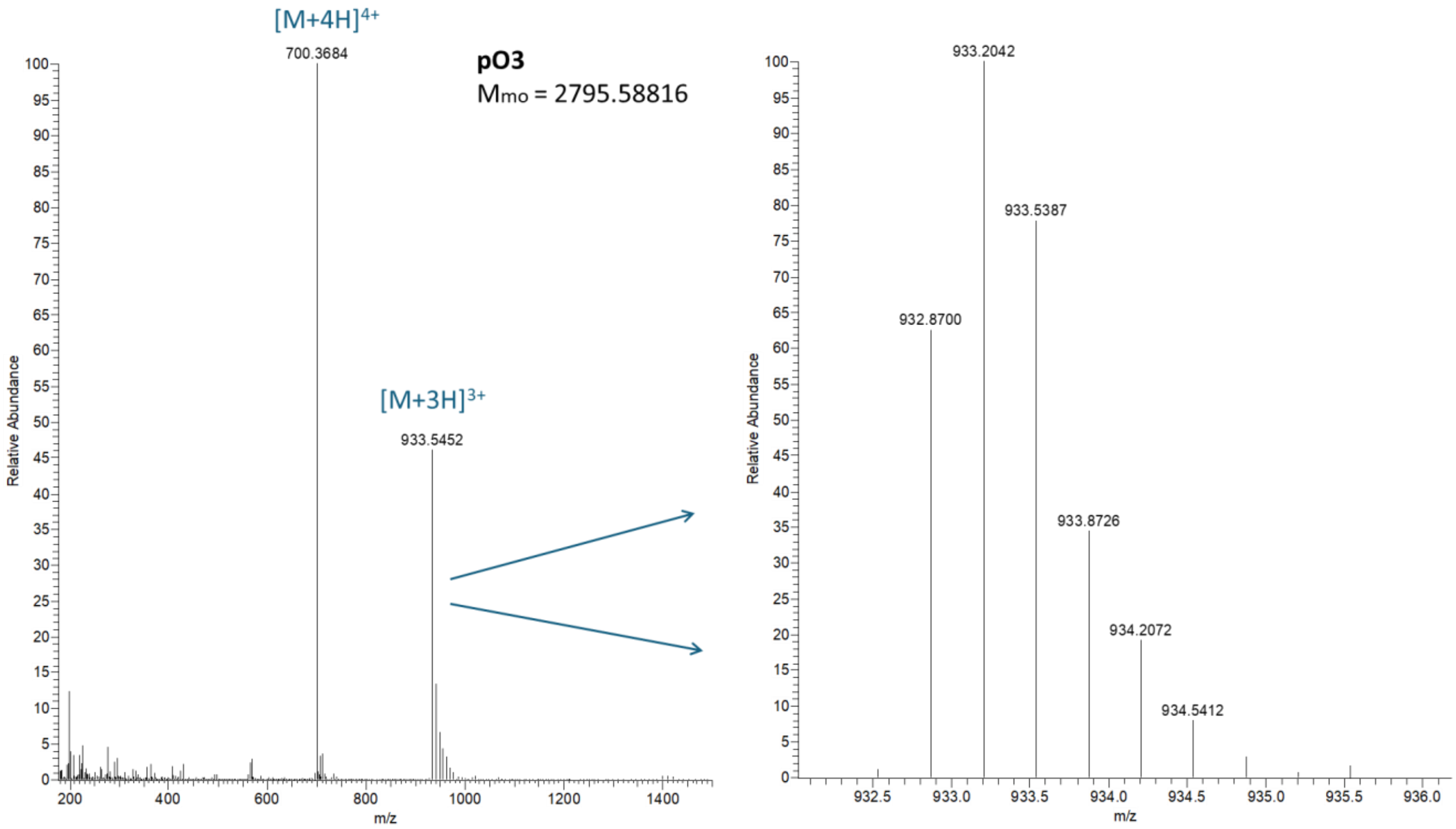

Fig. S3. High-resolution mass spectrum, measured on a Thermo Scientific Q Exactive Focus Hybrid Quadrupole-Orbitrap Mass Spectrometer (Thermo Fisher Scientific Inc.). Full *m/z* range and the magnification of the peak corresponding to the $[M+3H]^{3+}$ ion.

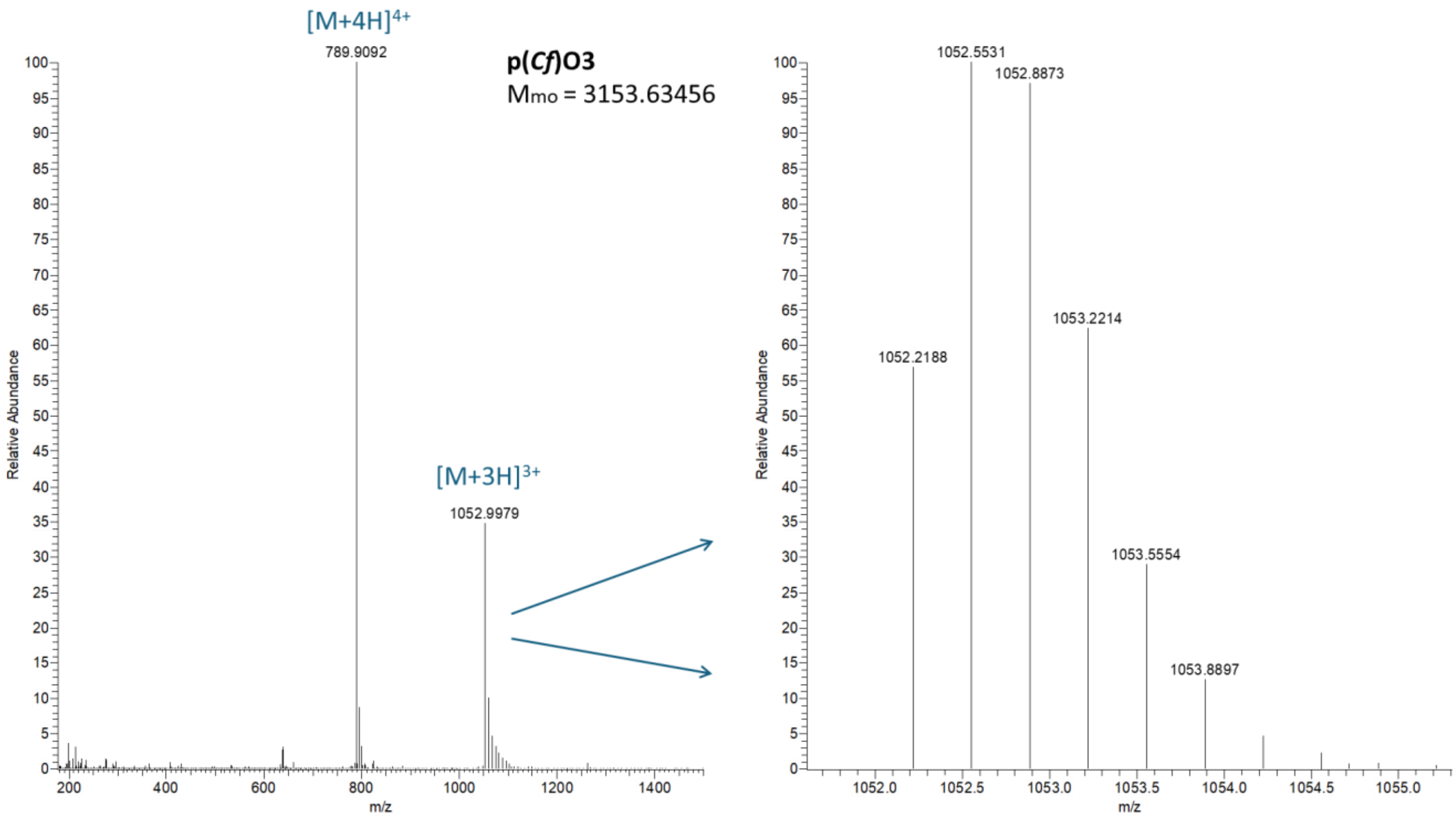

Fig. S4. High-resolution mass spectrum, measured on a Thermo Scientific Q Exactive Focus Hybrid Quadrupole-Orbitrap Mass Spectrometer (Thermo Fisher Scientific Inc.). Full *m/z* range and the magnification of the peak corresponding to the $[M+3H]^{3+}$ ion.

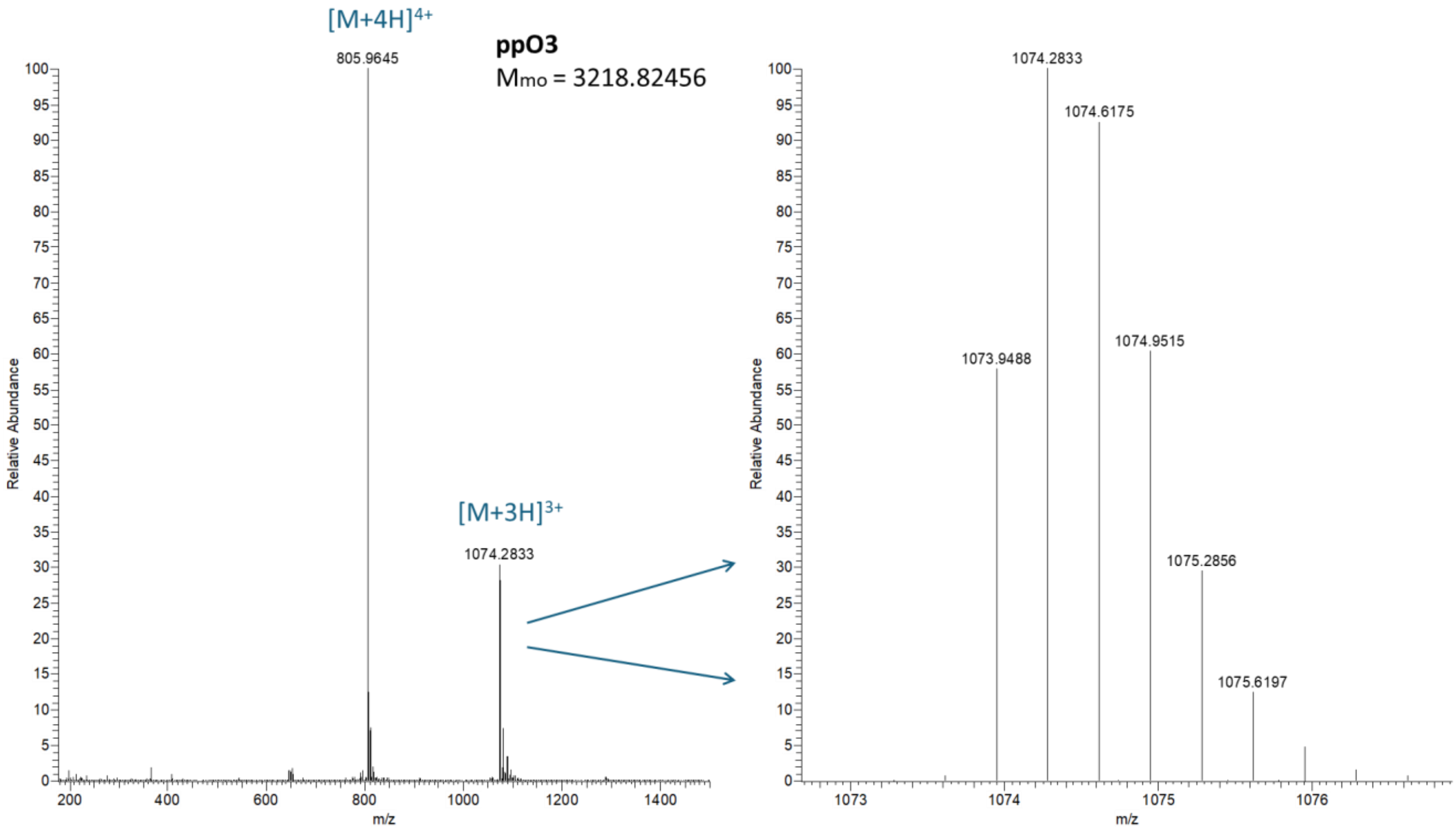

Fig. S5. High-resolution mass spectrum, measured on a Thermo Scientific Q Exactive Focus Hybrid Quadrupole-Orbitrap Mass Spectrometer (Thermo Fisher Scientific Inc.). Full *m/z* range and the magnification of the peak corresponding to the $[M+3H]^{3+}$ ion.

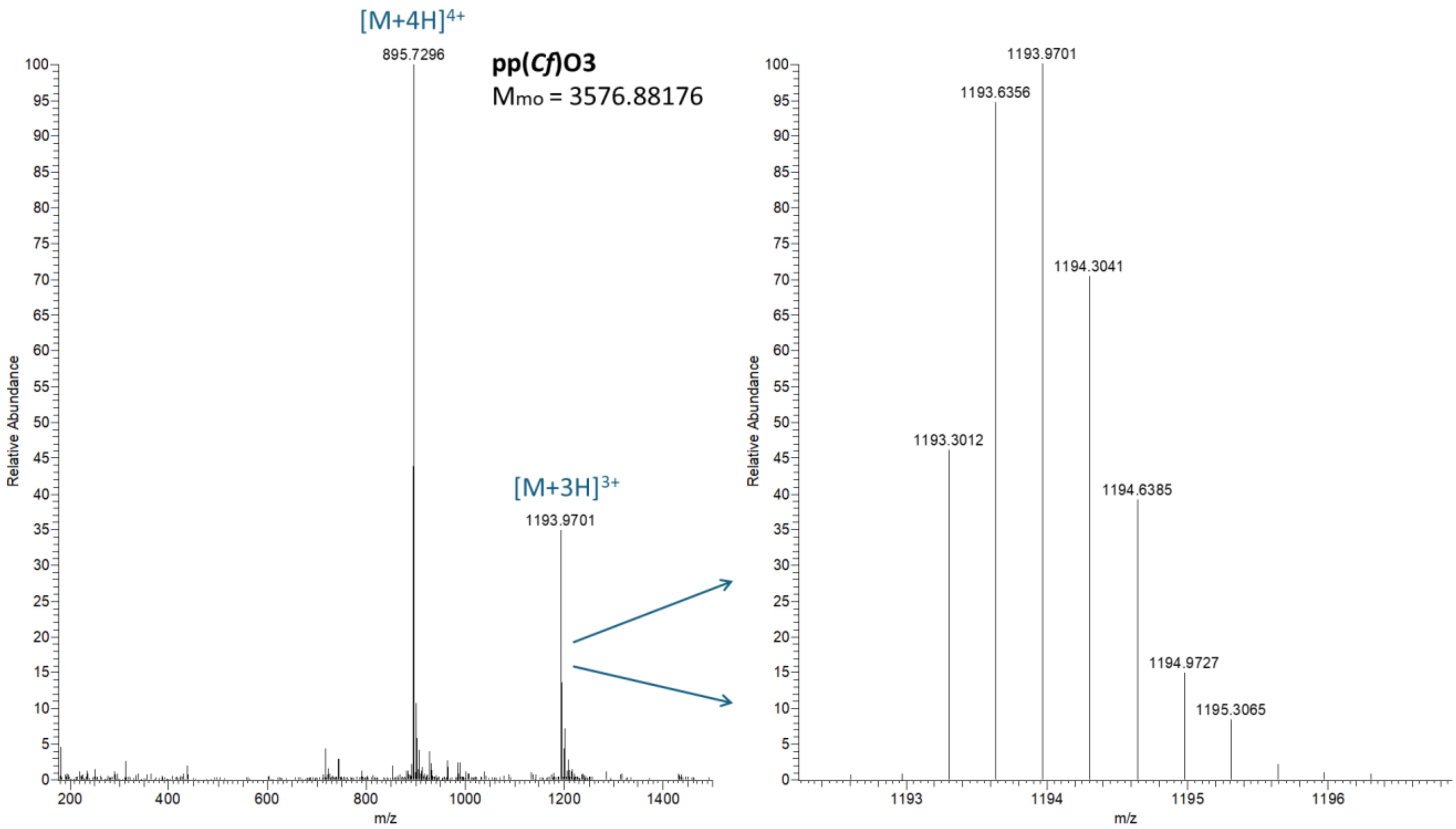

Fig. S6. High-resolution mass spectrum, measured on a Thermo Scientific Q Exactive Focus Hybrid Quadrupole-Orbitrap Mass Spectrometer (Thermo Fisher Scientific Inc.). Full *m/z* range and the magnification of the peak corresponding to the $[M+3H]^{3+}$ ion.

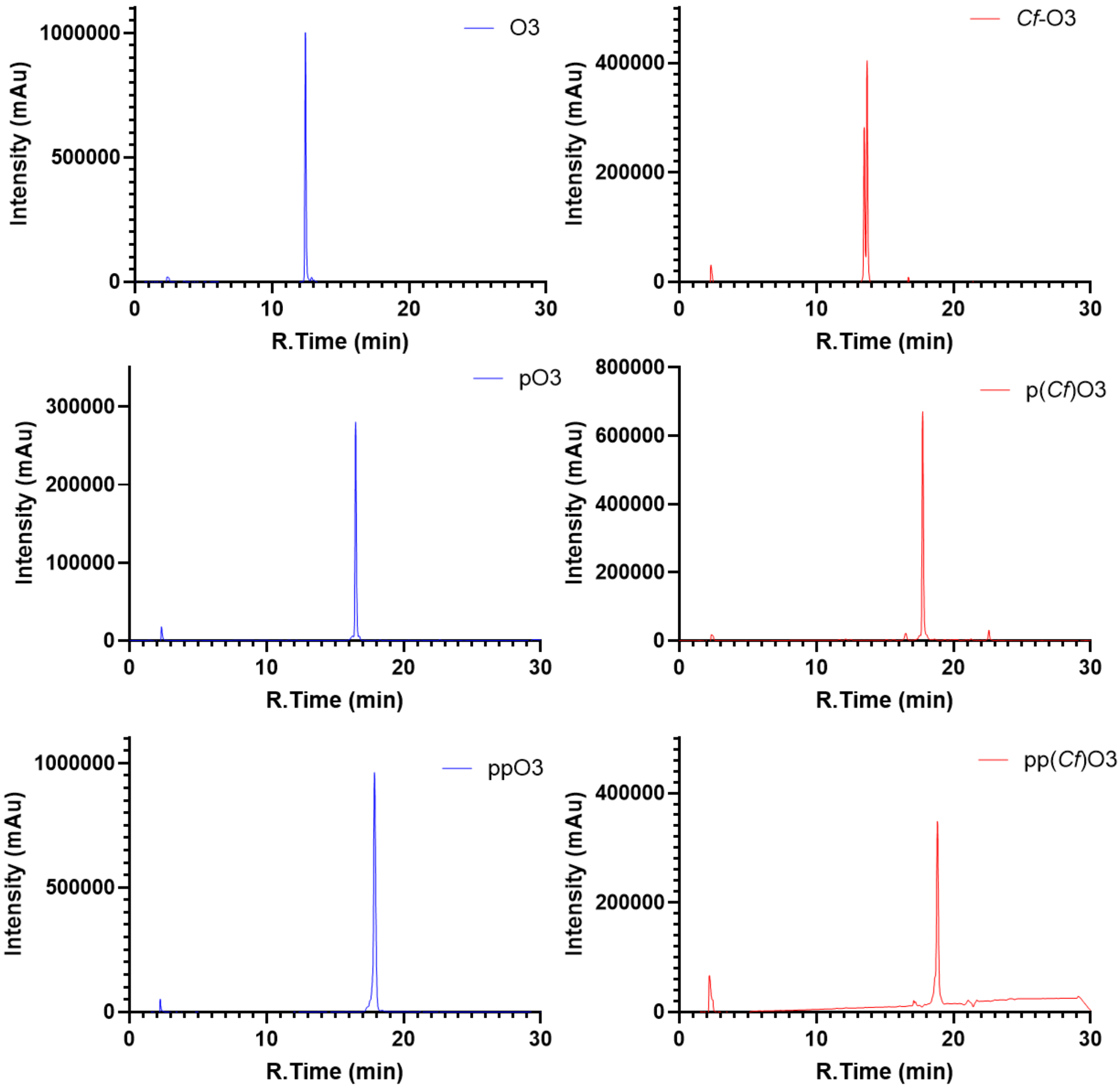

Fig. S7. RP-HPLC chromatograms of the peptides, measured on a Shimadzu LC-40 system, using a Phenomenex Jupiter C12 column, detected at 220 nm. The gradient was 5–100% B, 20 min. In the case of CfO3 peptide, the two isomers, corresponds to the 5(6) isomers of carboxyfluorescein, eluted as two peaks on the HPLC chromatogram.

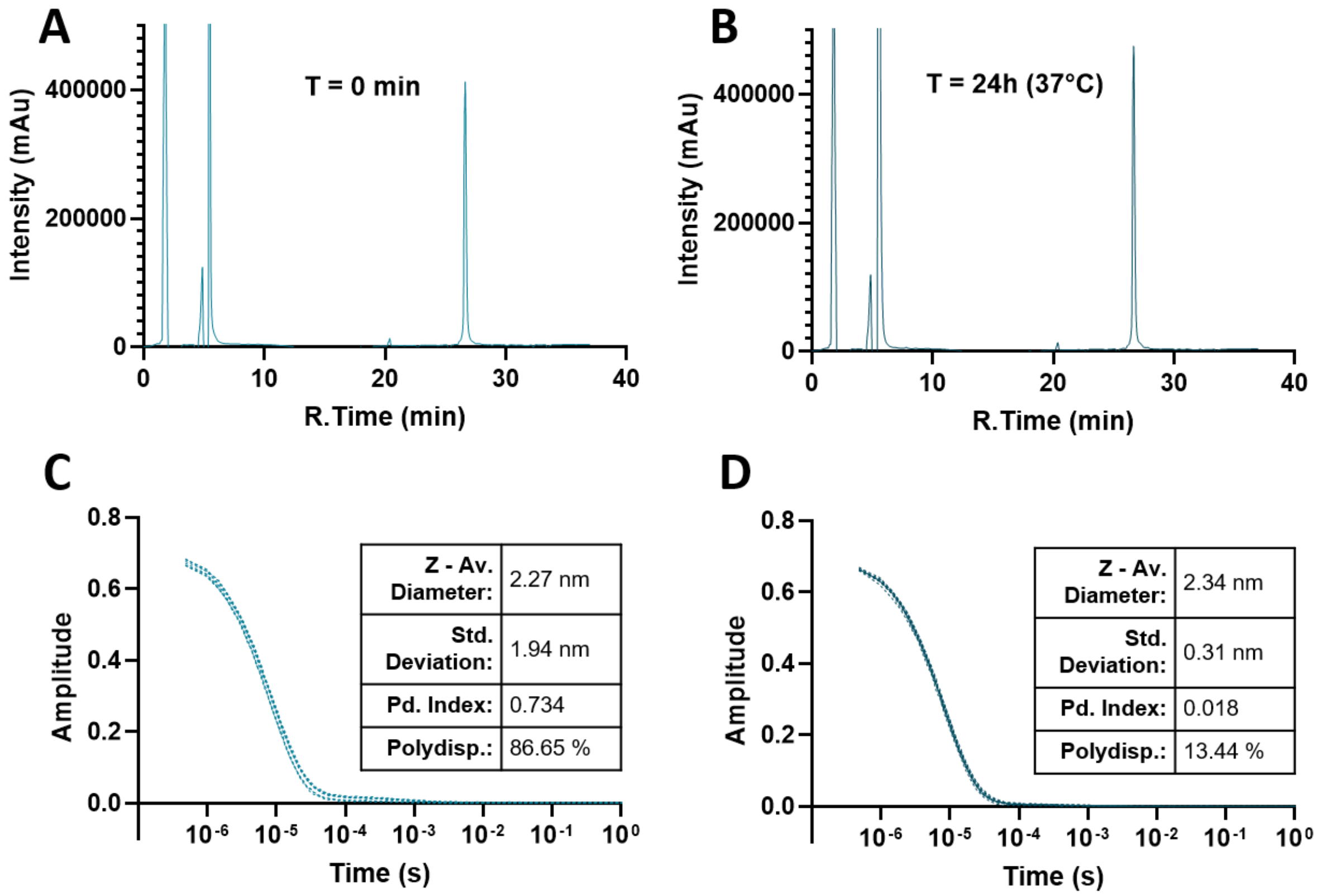

Fig. S8. Stability and size distribution of the ppO3+CyD system. RP-HPLC chromatograms of the peptides were recorded 10 min after solubilisation of the ppO3 peptide in CyD/PBS solution (**A**) and after 24 hours of agitation at 37 °C (**B**). Shimadzu LC-40 system was used with a ReproShell ODS-1, 5 µm, 250 x 4.6 mm column (Dr. Maisch High Performance LC GmbH, Ammerbuch-Entringen Germany). The gradient was 5–100% B, 30 min. To visualize the size distribution and stability of the ppO3+CyD complex, dynamic light scattering (DLS) was performed using a W130i DLS instrument using low-volume disposable cuvettes with 1 cm path length. The correlation functions (**C** and **D**) were measured without filtration at 20 °C for 10 s, repeated 10 times and averaged. Data analysis to determine the average hydrodynamic diameter together with the standard deviation and the polydispersity, was performed using the iSize 3.0 software. Panel **C** shows the results recorded 10 min after dissolution, while panel **D** was recorded after 24 hours of agitation at 37 °C.

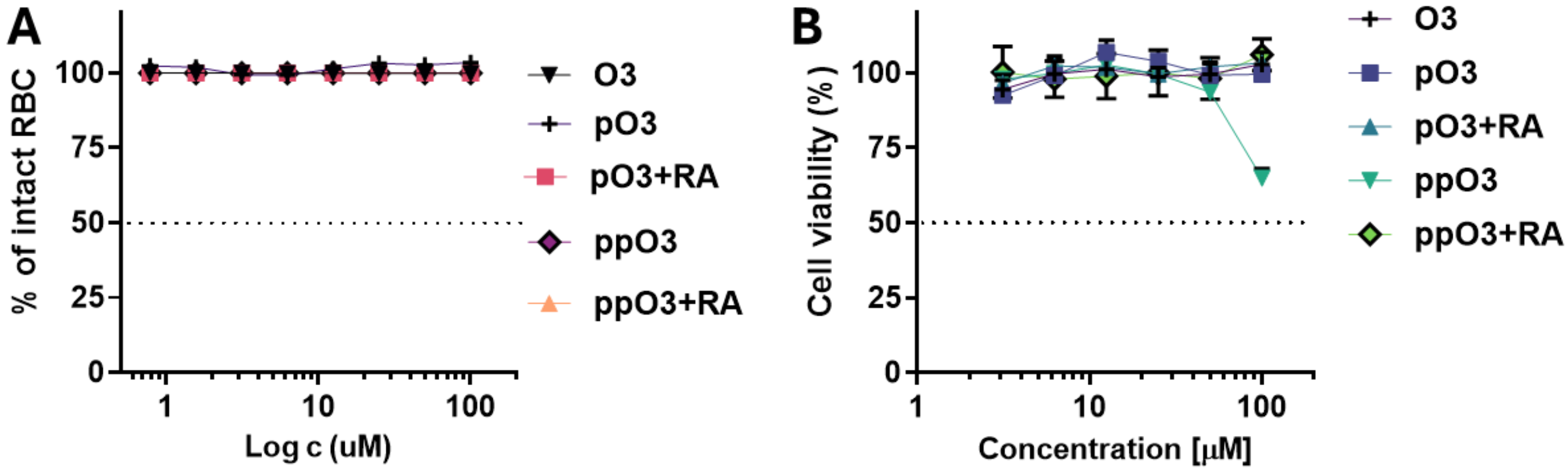

Fig. S9. *In vitro* toxicity of the compounds**.** Hemolytic activity (**A**) was assayed using human red blood cells (1% (*V/V*)) and the percentage of hemolysis, compared to medium-treated control wells, was plotted. Cytotoxicity (**B**) of the compounds was measured on HEK-293 human embryonic kidney cells after 4 hours of incubation, using MTT viability assay. All data are presented as mean ± SD (*N = 3*).

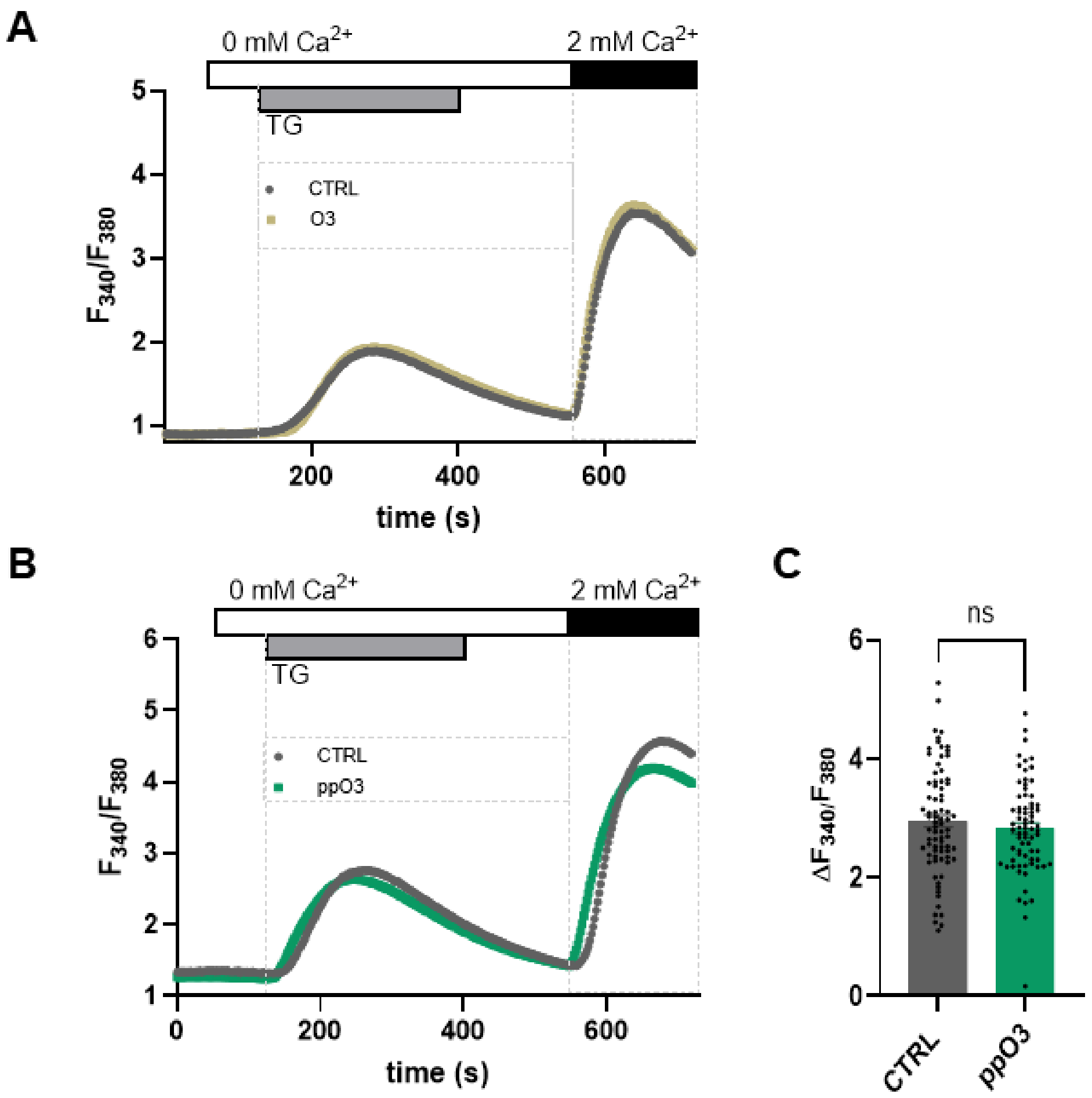

Fig. S10. Cytosolic calcium transients and SOCE amplitude in HEK293 cells pretreated with non-lipidated O3 or CyD-free ppO3. **(A)** Time course of cytosolic calcium responses after pretreatment with non-lipidated O3, which mainly localizes to endosomes and does not affect SOCE. **(B)** Time course of cytosolic calcium responses after pretreatment with CyD-free ppO3. **(C)** SOCE amplitude quantification showing no significant difference from untreated controls. Data are mean ± SEM; each dot represents one cell from three independent experiments with different cell preparations; statistical significance was assessed by Student's t-test.
